# Supplementary material for: Association of membership in a farmer producer organization with crop diversity, household income, diet diversity, and women’s empowerment in Uttar Pradesh, India
Source: PLoS One. 2025 Mar 26;20(3):e0319704. doi: 10.1371/journal.pone.0319704 (PMC11940710; doi:10.1371/journal.pone.0319704)
Supplement: S1 Text — (DOCX) [file pone.0319704.s002.docx]

| **Table 1.** Summary of missing data.^1^ | |
| --- | --- |
| Household members | 0% (0) |
| Age | 0% (0) |
| Sex | 0% (0) |
| Educational attainment: Male | 0% (0) |
| Educational attainment: Female | 2.3% (19) |
| Caste | <0.1% (1) |
| Farm size | 0% (0) |
| Agricultural land owned | 1.7% (14) |
| Cultivated crops | 0% (0) |
| Total land cultivated in the kharif season | 0% (0) |
| Types of livestock owned | 0% (0) |
| Had income from cultivation | 0% (0) |
| Cultivation income (amount) | 0% (0) |
| Had income from livestock | 0% (0) |
| Livestock income (amount) | 0% (0) |
| Had income from wages | 0% (0) |
| Wage income (amount) | 0% (0) |
| Had income from non-agriculture source | 0% (0) |
| Other income (amount) | 0% (0) |
| Diet diversity | 0% (0) |
| Women’s empowerment | 0% (0) |
| ^1^Values are % (n). | |

| **Table 2.** Association between farmer producer organization (FPO) participation and crop diversity (n=809 households).^1^ | | | | |
| --- | --- | --- | --- | --- |
|  | Dependent variable:  Count of crops cultivated | | | |
| **Independent variable** | **Beta** | **SE** | **95% CI** | **p-value** |
| **FPO household** | 0.30 | 0.104 | 0.09, 0.50 | 0.005 |
| **Caste** |  |  |  |  |
| Scheduled Caste/Tribe | Ref |  |  | <0.001 |
| Other Backward Caste | 0.20 | 0.137 | -0.07, 0.47 |  |
| General/Other | -0.64 | 0.144 | -0.92, -0.36 |  |
| **Educational Attainment: Male** |  |  |  |  |
| No formal school (including illiterate) | Ref |  |  | 0.075 |
| Primary, complete (grade 1-4) | 0.38 | 0.192 | 0.00, 0.75 |  |
| High school, complete (grade 5-9) | 0.12 | 0.176 | -0.23, 0.46 |  |
| Secondary school, complete | 0.10 | 0.207 | -0.31, 0.51 |  |
| Graduate and above | -0.15 | 0.229 | -0.60, 0.30 |  |
| **Educational Attainment: Female** |  |  |  |  |
| No formal school (including illiterate) | Ref |  |  | 0.004 |
| Primary, complete (grade 1-4) | -0.43 | 0.131 | -0.69, -0.17 |  |
| High school, complete (grade 5-9) | -0.33 | 0.144 | -0.61, -0.05 |  |
| Secondary school, complete | -0.16 | 0.206 | -0.56, 0.24 |  |
| Graduate and above | 0.40 | 0.326 | -0.24, 1.0 |  |
| **Total land cultivated in the kharif season (hectares)** | 0.67 | 0.737 | -0.77, 2.1 | <0.001 |
| Abbreviations: CI, confidence interval; SE, standard error.  ^1^Model was estimated using multivariable linear regression. | | | | |

| **Table 3.** Association between farmer producer organization (FPO) participation and household monthly income (n=790 households).^1^ | | | | |
| --- | --- | --- | --- | --- |
|  | Dependent variable:  Total monthly household income | | | |
| **Independent variables** | **Marginal effects** | **SE** | **95% CI** | **p-value** |
| **FPO household** | 601.9 | 242.0 | 127.5, 1,076 | 0.013 |
| **Caste** |  |  |  |  |
| Scheduled Caste/Tribe | Ref |  |  |  |
| Other Backward Caste | 1,377 | 279.4 | 829.1, 1,924 | <0.001 |
| General/Other | 701.7 | 315.2 | 83.88, 1,319 | 0.026 |
| **Educational Attainment: Male** |  |  |  |  |
| No formal school including (illiterate) | Ref |  |  |  |
| Primary, complete grade (1-4 | -507.6 | 437.2 | -1,365, 349.4 | 0.246 |
| High school, complete grade (5-9) | -697.5 | 490.4 | -1,659, 263.6 | 0.155 |
| Secondary school, complete | -765.2 | 453.0 | -1,653, 122.7 | 0.091 |
| Graduate and above | -243.6 | 520.3 | -1,263, 776.2 | 0.640 |
| **Educational Attainment: Female** |  |  |  |  |
| No formal school including illiterate |  |  |  |  |
| Primary, complete grade (1-4) | -2,581 | 1,254 | -5,038, -123.7 | 0.040 |
| High school, complete grade (5-9) | -2,597 | 1,255 | -5,056, -138.7 | 0.039 |
| Secondary school, complete | -3,392 | 1,266 | -5,873, -912.1 | 0.007 |
| Graduate and above | -2,288 | 1,288 | -4,813, 237.0 | 0.076 |
| **Household members** | 265.6 | 64.14 | 139.9, 391.3 | <0.001 |
| **Total land cultivated in the kharif season (hectares)** | 1,975 | 261.4 | 1,463, 2,487 | <0.001 |
| Abbreviations: CI, confidence interval; SE, standard error.  ^1^The dependent variable is the total household income monthly across income sources. Model was estimated with a two-part linear estimator. Coefficient estimates are shown as marginal differences with robust, heteroskedasticity-consistent standard errors. | | | | |

| **Table 4.** Association between farmer producer organization (FPO) participation and having a diverse diet (n=1609).^1^ | | | | |
| --- | --- | --- | --- | --- |
|  | Dependent variable:  Diet diversity score ≥5 | | | |
| **Independent variables** | **AOR** | **SE** | **95% CI** | **p-value** |
| **FPO household** | 1.35 | 0.137 | 1.08, 1.68 | 0.007 |
| **Caste** |  |  |  |  |
| Scheduled Caste/Tribe | Ref |  |  | <0.001 |
| Other Backward Caste | 1.04 | 0.169 | 0.80, 1.37 |  |
| General/Other | 0.54 | 0.222 | 0.38, 0.76 |  |
| **Educational Attainment** |  |  |  |  |
| No formal school (including illiterate) | Ref |  |  | <0.001 |
| Primary, complete (grade 1-4) | 0.45 | 0.161 | 0.33, 0.61 |  |
| High school, complete (grade 5-9) | 0.59 | 0.164 | 0.44, 0.79 |  |
| Secondary school, complete | 0.76 | 0.199 | 0.53, 1.07 |  |
| Graduate and above | 0.61 | 0.245 | 0.39, 0.94 |  |
| **Total land cultivated in the kharif season (hectares)** | 1.35 | 0.117 | 1.14, 1.62 | <0.001 |
| **Sex** |  |  |  |  |
| Female | Ref |  |  | 0.029 |
| Male | 1.28 | 0.080 | 1.03, 1.61 |  |
| Abbreviations: AOR, Adjusted Odds Ratio; CI, Confidence Interval; SE, Standard Error.  ^1^Model was estimated using multivariable logistic regression. Standard error accounts for clustering within households. | | | | |

| **Table 5.** Association between farmer producer organization (FPO) participation and diet diversity score (n=1609).^1^ | | | | |
| --- | --- | --- | --- | --- |
|  | Dependent variable:  Diet diversity score (range: 0 to 10) | | | |
| **Independent variables** | **Beta** | **SE** | **95% CI** | **p-value** |
| **FPO household** | 0.28 | 0.066 | 0.15, 0.41 | <0.001 |
| **Caste** |  |  |  |  |
| Scheduled Caste/Tribe | Ref |  |  | 0.50 |
| Other Backward Caste | -0.09 | 0.094 | -0.27, 0.10 |  |
| General/Other | -0.11 | 0.101 | -0.31, 0.09 |  |
| **Educational Attainment** |  |  |  |  |
| No formal school (including illiterate) | Ref |  |  | 0.015 |
| Primary, complete (grade 1-4) | -0.29 | 0.088 | -0.46, -0.12 |  |
| High school, complete (grade 5-9) | -0.24 | 0.093 | -0.42, -0.05 |  |
| Secondary school, complete | -0.27 | 0.120 | -0.50, -0.03 |  |
| Graduate and above | -0.30 | 0.143 | -0.59, -0.02 |  |
| **Total land cultivated in the kharif season (hectares)** | 0.22 | 0.061 | 0.10, 0.34 | <0.001 |
| **Sex** |  |  |  |  |
| Female | Ref |  |  | 0.016 |
| Male | 0.16 | 0.068 | 0.03, 0.30 |  |
| Abbreviations: CI, Confidence Interval; SE, Standard Error.  ^1^Model was estimated using multivariable linear regression. Standard error accounts for clustering within households. | | | | |

| **Table 6.** Dietary intake of adults participating in a cross-sectional survey, by caste, comparing households with and without farmer producer organization (FPO) members in Uttar Pradesh, India.^1^ | | | | | | |
| --- | --- | --- | --- | --- | --- | --- |
|  | **Scheduled Caste/Tribe** | | **Other Backward Caste** | | **General/Other** | |
|  | **FPO household** n=142^1^ | **Non-FPO household** n=177^1^ | **FPO household** n=580^1^ | **Non-FPO household** n=363^1^ | **FPO household** n=103^1^ | **Non-FPO household** n=243^1^ |
| **Minimum dietary diversity score** | 4.5 (1.5) | 3.9 (1.4) | 4.2 (1.4) | 4.0 (1.2) | 4.0 (0.9) | 4.0 (1.0) |
| **Diverse diet (minimum dietary diversity score ≥5)** | 47% (67) | 31% (54) | 44% (258) | 34% (123) | 18% (19) | 27% (65) |
| **Nutritious foods** |  |  |  |  |  |  |
| Gourds | 35% (49) | 15% (26) | 27% (158) | 11% (40) | 13% (13) | 14% (35) |
| Cucumber, capsicum, drumstick | 32% (46) | 19% (33) | 31% (178) | 18% (67) | 16% (16) | 20% (49) |
| Green Leafy: mustard, spinach, other | 13% (18) | 8.5% (15) | 9.3% (54) | 9.6% (35) | 3.9% (4) | 2.5% (6) |
| Papaya, mango | 33% (47) | 11% (20) | 23% (133) | 12% (42) | 16% (16) | 25% (61) |
| Banana, apple, watermelon | 27% (39) | 8.5% (15) | 18% (104) | 5.8% (21) | 6.8% (7) | 6.6% (16) |
| Grapes, peaches, jackfruit | 30% (43) | 6.2% (11) | 22% (130) | 8.8% (32) | 8.7% (9) | 6.2% (15) |
| Paneer | 5.6% (8) | 0% (0) | 1.6% (9) | 1.1% (4) | 1.0% (1) | 0.8% (2) |
| Curd | 42% (60) | 25% (44) | 39% (225) | 34% (125) | 20% (21) | 10% (25) |
| Milk | 54% (76) | 50% (88) | 48% (281) | 48% (174) | 73% (75) | 80% (195) |
| Fish | 21% (12) | 34% (29) | 46% (85) | 31% (58) | 0% (0) | 0% (0) |
| Peanuts, cashews, almonds, pistachios, walnuts, pumpkin seeds, or sunflower seeds | 1.4% (2) | 3.4% (6) | 1.4% (8) | 0.8% (3) | 1.9% (2) | 1.6% (4) |
| **Unhealthy foods** |  |  |  |  |  |  |
| Cake, biscuits, halwa, jalebi, ladoo | 61% (86) | 45% (80) | 59% (344) | 47% (171) | 66% (68) | 59% (143) |
| Other mithai, kulfi, ice cream, shakes | 15% (22) | 1.7% (3) | 17% (96) | 5.0% (18) | 4.9% (5) | 4.9% (12) |
| Chips, namkeen | 6.3% (9) | 2.8% (5) | 11% (63) | 3.0% (11) | 13% (13) | 2.5% (6) |
| Maggi noodles, wai wai | 18% (25) | 4.0% (7) | 13% (74) | 5.8% (21) | 3.9% (4) | 2.9% (7) |
| Samosa, pakora, puri, vada | 63% (89) | 32% (57) | 41% (239) | 30% (109) | 71% (73) | 55% (134) |
| Fruit juice, frooti | 14% (20) | 7.9% (14) | 10% (60) | 4.7% (17) | 3.9% (4) | 2.5% (6) |
| Cold drinks | 35% (49) | 14% (25) | 21% (123) | 13% (46) | 16% (16) | 8.6% (21) |
| ^1^Values are mean (SD) or % (n). | | | | | | |

| **Table 7.** Association between farmer producer organization (FPO) participation and women’s empowerment (n= 789).^1^ | | | | |
| --- | --- | --- | --- | --- |
|  | Dependent variable:  Overall women’s empowerment^2^ | | | |
| **Independent variables** | **AOR** | **SE** | **95% CI** | **p-value** |
| **FPO household** | 0.96 | 0.151 | 0.72, 1.30 | 0.81 |
| **Caste** |  |  |  |  |
| Scheduled Caste/Tribe | Ref |  |  | 0.011 |
| Other Backward Caste | 0.98 | 0.187 | 0.67, 1.42 |  |
| General/Other | 1.71 | 0.233 | 1.09, 2.71 |  |
| **Educational Attainment** |  |  |  |  |
| No formal school (including illiterate) | Ref |  |  | 0.23 |
| Primary, complete (grade 1-4) | 1.17 | 0.190 | 0.81, 1.70 |  |
| High school, complete (grade 5-9) | 0.90 | 0.189 | 0.62, 1.30 |  |
| Secondary school, complete | 1.54 | 0.260 | 0.93, 2.59 |  |
| Graduate and above | 0.71 | 0.426 | 0.29, 1.65 |  |
| **Total land cultivated in the kharif season (hectares)** | 0.74 | 0.134 | 0.57, 0.95 | 0.018 |
| Abbreviations: AOR, adjusted odds ratio; CI, Confidence Interval; SE, Standard Error.  ^1^Model was estimated using multivariable logistic regression.  ^2^Overall empowerment is achieved when a woman is empowered in at least three out of the five domains. Domains included (1) input in productive decisions (“production”); (2) ownership of assets and access to and decisions about credit (together, “resources”); (3) control over use of income (“income”); (4) self-help group membership (“leadership”); and (5) workload (“time balance”). | | | | |

| **Table 8.** Indicators of women’s empowerment among women participating in a cross-sectional survey, by caste, comparing households with and without farmer producer organization (FPO) members in Uttar Pradesh, India.^1^ | | | | | | |
| --- | --- | --- | --- | --- | --- | --- |
|  | **Scheduled Caste/Tribe** | | **Other Backward Caste** | | **General/Other** | |
|  | **FPO household**  N = 71^1^ | **Non-FPO household**  N = 92^1^ | **FPO household**  N = 289^1^ | **Non-FPO household**  N = 176^1^ | **FPO household**  N = 51^1^ | **Non-FPO household**  N = 120^1^ |
| **Overall women’s empowerment**^2^ | 52% (37) | 46% (42) | 47% (135) | 49% (86) | 57% (29) | 63% (76) |
| **Women’s empowerment in agriculture indicators**^3^ |  |  |  |  |  |  |
| Input in productive decisions | 96% (68) | 95% (87) | 96% (276) | 91% (160) | 100% (51) | 98% (117) |
| Ownership of assets | 86% (61) | 99% (91) | 92% (266) | 93% (164) | 59% (30) | 72% (86) |
| Access to and decisions about credit | 1.4% (1) | 2.2% (2) | 4.2% (12) | 4.0% (7) | 0% (0) | 3.3% (4) |
| Control over use of income | 100% (71) | 100% (92) | 100% (289) | 98% (172) | 100% (51) | 98% (118) |
| Self-help group membership | 2.8% (2) | 0% (0) | 0.7% (2) | 1.1% (2) | 0% (0) | 0% (0) |
| Work balance | 52% (37) | 48% (44) | 47% (137) | 56% (98) | 57% (29) | 65% (78) |
| ^1^Values are % (n).  ^2^Overall empowerment is achieved when a woman is empowered in at least three out of the five domains.  ^3^Input in productive decisions: empowered if makes decisions or has input in decision in at least one area; Ownership of assets: empowered if solely or jointly owns at least one agricultural asset that is not a small agricultural asset; Access to and decisions about credit: empowered if solely/jointly make at least one decision regarding at least one source of credit; Control over use of income: empowered if has some input in how to use income and not only for minor household expenditures; Self-help group membership: empowered if actively participates in a self-help group; Work balance: empowered if time spent on productive tasks is less than 10.5 hours per day. | | | | | | |

| **Table 9**. Receipt of advice, training and inputs among farmer producer organization (FPO) households and non-FPO households.^1^ | | |
| --- | --- | --- |
| Variable | **FPO households** | **Non-FPO households** |
| **Received any regular farm-related advice** | 28% (114) | 1% (4) |
| **Source of advice** |  |  |
| FPO | 89% (101) | 0% (0) |
| Government or KVK (Krishi Vigyan Kendra) | 8% (9) | 100% (4) |
| **Among those who received any advice** | n=114 | n=4 |
| **Type of advice** |  |  |
| Weather | 92% (105) | 100% (4) |
| Pest incidence and management | 89% (102) | 75% (3) |
| Market prices | 84% (96) | 75% (3) |
| **Receive any training on crop production** | 19% (78) | 1% (2) |
| **Training source** |  |  |
| FPO | 97% (75) | 0% (0) |
| Government/KVK | 6% (5) | 0% (0) |
| **Purchased any inputs from FPO in past year^2^** | 10% (39) | - |
| **Among those who purchased inputs** | n=39 | - |
| **Inputs purchased^2^** |  |  |
| Fertilizer | 65% (26) | - |
| Pesticide | 80% (32) | - |
| Seeds | 65% (26) | - |
| Used any processing facilities | 0% (1) | - |
| ^1^Values are % (n),  ^2^ The questions on inputs purchased were asked only to FPO households. | | |
