# Supplementary material for: Association of membership in a farmer producer organization with crop diversity, household income, diet diversity, and women’s empowerment in Uttar Pradesh, India
Source: PLoS One. 2025 Mar 26;20(3):e0319704. doi: 10.1371/journal.pone.0319704 (PMC11940710; doi:10.1371/journal.pone.0319704)
Supplement: S1 File — (DOCX) [file pone.0319704.s003.docx]

| **PART A: INTERVIEW DETAILS** | | | | |
| --- | --- | --- | --- | --- |
| **NO.** | **VARIABLE NAME** | **QUESTION** | **OPTIONS** | **RESPONSE** |
| 1. | [Today_date] | Today’s date | DD/MM/YYYY |  |
| 2. |  | Interviewer ID | Text |  |
| 3. | [QA3] | Household ID | XXXX | [4 numeric digits] |
| 4. | [QA4] | Participant/Interviewee | Adult Male = 1  Adult Female = 2 |  |
| 5. | [QA5] | Type of household  *Note: Households will be FPO households in FPO villages and non-FPO households in remaining villages* | FPO household = 1  Non-FPO household = 2 |  |
| 6. | [QA6] | Village/Habitation name | Azampur Garwa = 1  Gauripur Budhwan = 2  Husenabad = 3  Lakhnipur = 4  Pure Rahat Ali = 5  Amauli = 6  Godainpur = 7  Argal = 8  Ashapur = 9  Roti = 10  Babai = 11  Babupur = 12  Baburihapur = 13  Bakiyapur = 14  Beeghanpur = 15  Bamthara = 16  Behta Khurd = 17  Bijauli = 18  Bilari = 19  Birnai = 20  Ranupur = 21  Budhwa = 22  Burhwan = 23  Chatanpur = 24  Madari = 25  Madri = 26  Makrandpur = 27  Dapsaura = 28  Devchali = 29  Mominpur = 30  Digharua = 31  Jajmoiya = 32  Kapil = 33  Kauh = 34  Paradhanai = 35  Kevtara Bharsa = 36  Kulkhera = 37  Madiha khera = 38  Mangalpur = 39  Mirjapur = 40  Naraicha = 41  Naseniya = 42  Nonara = 43  Nyori Jalalpur = 44  Paprenda = 45  Firojpur = 46  Rampur Hussaina/Husena = 47  Rampur Kurmi = 48  Salempur kanaira/Kanera/Bhagaunapur = 49  Sarahan Bujurg = 50  Sarhankhurd = 51  Husepur = 52  Khairabad = 53  Deori Bujurg = 54  Hasanpur Devari = 55  Pandepur = 56  Sahimalpur = 57 |  |
| **PART B: INCLUSION CRITERIA** | | | | |
| **NO.** | **VARIABLE NAME** | **QUESTION** | **OPTIONS** | **RESPONSE** |
| 1. | [QB1] | Are you or someone in this household engaged in agriculture defined as cultivating a crop on self-owned or leased land? | Yes = 1  No = 2 |  |
| 2. | [QB2] | What is your date of birth?  *If month is unknown, enter “07”. If day is unknown, enter “01”.* | DD/MM/YYYY |  |
| 3. | [QB3] | Participant’s age | XX years | [auto-calculate based on Q2] |
| 4. | [QB4] | Does participant meet the age criterion (>18 years old)? | Yes = 1  No = 2 | [auto-calculate based on Q3] |
| **Participant is eligible if Q1 & 4 are ‘YES’** | | | | |
| 5. | [QB5] | Is participant eligible? | Yes = 1  No = 2 [end survey] | [auto-calculate based on Q1-4] |
| 6. | [QB6] | Is the participant willing to participate in the study?  *Hint: Check if a woman in the household is willing to talk, if not they are not eligible.* | Yes = 1 [Skip to Q9]  No = 2 |  |
| 7. | [QB7] | Why are you not willing to participate in the study? | No time = 1 [end survey]  Not interested = 2 [end survey]  Women in the household will not talk to enumerator = 3 [end survey]  Other = 4 [answer Q8] |  |
| 8. | [QB8] | Specify other reason for not being willing to participate. | Text [end survey] |  |
| 9. |  | Name of respondent | Text |  |
| 10. |  | Phone number | Number |  |
| **PART C: DEMOGRAPHICS** | | | | |
| **NO.** | **VARIABLE NAME** | **QUESTION** | **OPTIONS** | **RESPONSE** |
| 1. | [QC1] | What is your current marital status? | Currently married = 1  Widowed = 2  Divorced /Separated = 3  Never married = 4 |  |
| 2. | [QC2] | What is your relation to the other survey participant from your household? | Husband/Wife = 1  Father/Mother = 2  Son/Daughter = 3  Mother-in-law/Father-in-law = 4  Son-in-law/Daughter-in-law = 5  Other = 6 |  |
| 3. | [QC3] | What is the highest level of education that you have achieved?  *Hint: illiterate is a person who can neither read nor write or can only read but cannot write in any language.* | Illiterate = 1  Literate, no formal schooling = 2  Primary education (up to class IV) = 3  High School (class V to IX) = 4  Secondary school/Intermediary (ITI course, class X to XII/ or Intermediate) = 5  Graduate (BA, BSc, B.Com/Diploma) = 6  Professional degree/post graduate = 7  Other = 8 |  |
| 4. | [QC4] | Would it be alright for me to ask, what is your caste group? | Scheduled Caste (SC) = 1  Scheduled Tribe (ST) = 2  Other Backward Caste (OBC) = 3  General/Other = 4  Muslim (OBC) = 5  Muslim (General) = 6  No caste/tribe = 7  Other = 8  Prefer not to answer = 9 |  |
| \| **PART D: TIME USE** \| \| \| \| \| \| \| \| \| \| \| \| \| \| --- \| --- \| --- \| --- \| --- \| --- \| --- \| --- \| --- \| --- \| --- \| --- \| --- \| \| Record a log of the activities for the individual in the last complete 24 hours (from yesterday morning 4 AM to today morning 4 AM). The time intervals are marked in 30-minute intervals and one activity can be marked for each time period. \| \| \| \| \| \| \| \| \| \| \| \| \| \| VARIABLE NAME \| 4:00 to 4:30 AM_Part_D_timeuse \| 4:30 to 5:00 AM_Part_D_timeuse \| 5:00 to 5:30 AM_Part_D_timeuse \| 5:30 to 6:00 AM_Part_D_timeuse \| 6:00 to 6:30 AM_Part_D_timeuse \| 6:30 to 7:00 AM_Part_D_timeuse \| 7:00 to 7:30 AM_Part_D_timeuse \| 7:30 to 8:00 AM_Part_D_timeuse \| 8:00 to 8:30 AM_Part_D_timeuse \| 8:30 to 9:00 AM_Part_D_timeuse \| 9:00 to 9:30 AM_Part_D_timeuse \| 9:30 to 10:00 AM_Part_D_timeuse \| \| Time \| 4:00 to 4:30 AM \| 4:30 to 5:00 AM \| 5:00 to 5:30 AM \| 5:30 to 6:00 AM \| 6:00 to 6:30 AM \| 6:30 to 7:00 AM \| 7:00 to 7:30 AM \| 7:30 to 8:00 AM \| 8:00 to 8:30 AM \| 8:30 to 9:00 AM \| 9:00 to 9:30 AM \| 9:30 to 10:00 AM \| \| 1. Resting \|  \|  \|  \|  \|  \|  \|  \|  \|  \|  \|  \|  \| \| 2. Eating \|  \|  \|  \|  \|  \|  \|  \|  \|  \|  \|  \|  \| \| 3. Personal care \|  \|  \|  \|  \|  \|  \|  \|  \|  \|  \|  \|  \| \| 4. Cooking \|  \|  \|  \|  \|  \|  \|  \|  \|  \|  \|  \|  \| \| 5. Domestic work \|  \|  \|  \|  \|  \|  \|  \|  \|  \|  \|  \|  \| \| 6. Family care \|  \|  \|  \|  \|  \|  \|  \|  \|  \|  \|  \|  \| \| 7. Farming \|  \|  \|  \|  \|  \|  \|  \|  \|  \|  \|  \|  \| \| 8. Agricultural labour for wages \|  \|  \|  \|  \|  \|  \|  \|  \|  \|  \|  \|  \| \| 9. Non-Agricultural labour for wages /Employed work \|  \|  \|  \|  \|  \|  \|  \|  \|  \|  \|  \|  \| \| 10. Self-employed work \|  \|  \|  \|  \|  \|  \|  \|  \|  \|  \|  \|  \| \| 11. Watching TV/reading/ Mobile \|  \|  \|  \|  \|  \|  \|  \|  \|  \|  \|  \|  \| \| 12. Social activities \|  \|  \|  \|  \|  \|  \|  \|  \|  \|  \|  \|  \| \| 13. Shopping \|  \|  \|  \|  \|  \|  \|  \|  \|  \|  \|  \|  \| \| 14. Travelling \|  \|  \|  \|  \|  \|  \|  \|  \|  \|  \|  \|  \| \| 15. Exercising \|  \|  \|  \|  \|  \|  \|  \|  \|  \|  \|  \|  \| \| 16. School/ Newspaper \|  \|  \|  \|  \|  \|  \|  \|  \|  \|  \|  \|  \| \| 17. Religious activities \|  \|  \|  \|  \|  \|  \|  \|  \|  \|  \|  \|  \| \| 18. Other \|  \|  \|  \|  \|  \|  \|  \|  \|  \|  \|  \|  \|  \| \| **PART D: TIME USE** \| \| \| \| \| \| \| \| \| \| \| \| \| \| --- \| --- \| --- \| --- \| --- \| --- \| --- \| --- \| --- \| --- \| --- \| --- \| --- \| \| Record a log of the activities for the individual in the last complete 24 hours (from yesterday morning 4 AM to today morning 4 AM). The time intervals are marked in 30-minute intervals and one activity can be marked for each time period. \| \| \| \| \| \| \| \| \| \| \| \| \| \| VARIABLE NAME \| 10:00 to 10:30 AM_Part_D_timeuse \| 10:30 to 11:00 AM_Part_D_timeuse \| 11:00 to 11:30 AM_Part_D_timeuse \| 11:30 to 12:00 PM_Part_D_timeuse \| 12:00 to 12:30 PM_Part_D_timeuse \| 12:30 to 1:00 PM_Part_D_timeuse \| 1:00 to 1:30 PM_Part_D_timeuse \| 1:30 to 2:00 PM_Part_D_timeuse \| 2:00 to 2:30 PM_Part_D_timeuse \| 2:30 to 3:00 PM_Part_D_timeuse \| 3:00 to 3:30 PM_Part_D_timeuse \| 3:30 to 4:00 PM_Part_D_timeuse \| \| Time \| 10:00 to 10:30 AM \| 10:30 to 11:00 AM \| 11:00 to 11:30 AM \| 11:30 to 12:00 PM \| 12:00 to 12:30 PM \| 12:30 to 1:00 PM \| 1:00 to 1:30 PM \| 1:30 to 2:00 PM \| 2:00 to 2:30 PM \| 2:30 to 3:00 PM \| 3:00 to 3:30 PM \| 3:30 to 4:00 PM \| \| Resting \|  \|  \|  \|  \|  \|  \|  \|  \|  \|  \|  \|  \| \| Eating \|  \|  \|  \|  \|  \|  \|  \|  \|  \|  \|  \|  \| \| Personal care \|  \|  \|  \|  \|  \|  \|  \|  \|  \|  \|  \|  \| \| Cooking \|  \|  \|  \|  \|  \|  \|  \|  \|  \|  \|  \|  \| \| Domestic work \|  \|  \|  \|  \|  \|  \|  \|  \|  \|  \|  \|  \| \| Family care \|  \|  \|  \|  \|  \|  \|  \|  \|  \|  \|  \|  \| \| Farming \|  \|  \|  \|  \|  \|  \|  \|  \|  \|  \|  \|  \| \| Agricultural labour for wages \|  \|  \|  \|  \|  \|  \|  \|  \|  \|  \|  \|  \| \| Non-Agricultural labour for wages /Employed work \|  \|  \|  \|  \|  \|  \|  \|  \|  \|  \|  \|  \| \| Self-employed work \|  \|  \|  \|  \|  \|  \|  \|  \|  \|  \|  \|  \| \| Watching TV/reading/ Mobile \|  \|  \|  \|  \|  \|  \|  \|  \|  \|  \|  \|  \| \| Social activities \|  \|  \|  \|  \|  \|  \|  \|  \|  \|  \|  \|  \| \| Shopping \|  \|  \|  \|  \|  \|  \|  \|  \|  \|  \|  \|  \| \| Travelling \|  \|  \|  \|  \|  \|  \|  \|  \|  \|  \|  \|  \| \| Exercising \|  \|  \|  \|  \|  \|  \|  \|  \|  \|  \|  \|  \| \| School/ College \|  \|  \|  \|  \|  \|  \|  \|  \|  \|  \|  \|  \| \| Religious activities \|  \|  \|  \|  \|  \|  \|  \|  \|  \|  \|  \|  \| \| Other \|  \|  \|  \|  \|  \|  \|  \|  \|  \|  \|  \|  \| \| \| --- \| --- \| --- \| --- \| --- \| --- \| --- \| --- \| --- \| --- \| --- \| --- \| --- \| --- \| --- \| --- \| --- \| --- \| --- \| --- \| --- \| --- \| --- \| --- \| --- \| --- \| --- \| --- \| --- \| --- \| --- \| --- \| --- \| --- \| --- \| --- \| --- \| --- \| --- \| --- \| --- \| --- \| --- \| --- \| --- \| --- \| --- \| --- \| --- \| --- \| --- \| --- \| --- \| --- \| --- \| --- \| --- \| --- \| --- \| --- \| --- \| --- \| --- \| --- \| --- \| --- \| --- \| --- \| --- \| --- \| --- \| --- \| --- \| --- \| --- \| --- \| --- \| --- \| --- \| --- \| --- \| --- \| --- \| --- \| --- \| --- \| --- \| --- \| --- \| --- \| --- \| --- \| --- \| --- \| --- \| --- \| --- \| --- \| --- \| --- \| --- \| --- \| --- \| --- \| --- \| --- \| --- \| --- \| --- \| --- \| --- \| --- \| --- \| --- \| --- \| --- \| --- \| --- \| --- \| --- \| --- \| --- \| --- \| --- \| --- \| --- \| --- \| --- \| --- \| --- \| --- \| --- \| --- \| --- \| --- \| --- \| --- \| --- \| --- \| --- \| --- \| --- \| --- \| --- \| --- \| --- \| --- \| --- \| --- \| --- \| --- \| --- \| --- \| --- \| --- \| --- \| --- \| --- \| --- \| --- \| --- \| --- \| --- \| --- \| --- \| --- \| --- \| --- \| --- \| --- \| --- \| --- \| --- \| --- \| --- \| --- \| --- \| --- \| --- \| --- \| --- \| --- \| --- \| --- \| --- \| --- \| --- \| --- \| --- \| --- \| --- \| --- \| --- \| --- \| --- \| --- \| --- \| --- \| --- \| --- \| --- \| --- \| --- \| --- \| --- \| --- \| --- \| --- \| --- \| --- \| --- \| --- \| --- \| --- \| --- \| --- \| --- \| --- \| --- \| --- \| --- \| --- \| --- \| --- \| --- \| --- \| --- \| --- \| --- \| --- \| --- \| --- \| --- \| --- \| --- \| --- \| --- \| --- \| --- \| --- \| --- \| --- \| --- \| --- \| --- \| --- \| --- \| --- \| --- \| --- \| --- \| --- \| --- \| --- \| --- \| --- \| --- \| --- \| --- \| --- \| --- \| --- \| --- \| --- \| --- \| --- \| --- \| --- \| --- \| --- \| --- \| --- \| --- \| --- \| --- \| --- \| --- \| --- \| --- \| --- \| --- \| --- \| --- \| --- \| --- \| --- \| --- \|  \| \| **PART D: TIME USE** \| \| \| \| \| \| \| \| \| \| \| \| \| \| --- \| --- \| --- \| --- \| --- \| --- \| --- \| --- \| --- \| --- \| --- \| --- \| --- \| \| Record a log of the activities for the individual in the last complete 24 hours (from yesterday morning 4 AM to today morning 4 AM). The time intervals are marked in 30-minute intervals and one activity can be marked for each time period. \| \| \| \| \| \| \| \| \| \| \| \| \| \| VARIABLE NAME \| 4:00 to 4:30 PM_Part_D_timeuse \| 4:30 to 5:00 PM_Part_D_timeuse \| 5:00 to 5:30 PM_Part_D_timeuse \| 5:30 to 6:00 PM_Part_D_timeuse \| 6:00 to 6:30 PM_Part_D_timeuse \| 6:30 to 7:00 PM_Part_D_timeuse \| 7:00 to 7:30 PM_Part_D_timeuse \| 7:30 to 8:00 PM_Part_D_timeuse \| 8:00 to 8:30 PM_Part_D_timeuse \| 8:30 to 9:00 PM_Part_D_timeuse \| 9:00 to 9:30 PM_Part_D_timeuse \| 9:30 to 10:00 PM_Part_D_timeuse \| \| Time \| 4:00 to 4:30 PM \| 4:30 to 5:00 PM \| 5:00 to 5:30 PM \| 5:30 to 6:00 PM \| 6:00 to 6:30 PM \| 6:30 to 7:00 PM \| 7:00 to 7:30 PM \| 7:30 to 8:00 PM \| 8:00 to 8:30 PM \| 8:30 to 9:00 PM \| 9:00 to 9:30 PM \| 9:30 to 10:00 PM \| \| Resting \|  \|  \|  \|  \|  \|  \|  \|  \|  \|  \|  \|  \| \| Eating \|  \|  \|  \|  \|  \|  \|  \|  \|  \|  \|  \|  \| \| Personal care \|  \|  \|  \|  \|  \|  \|  \|  \|  \|  \|  \|  \| \| Cooking \|  \|  \|  \|  \|  \|  \|  \|  \|  \|  \|  \|  \| \| Domestic work \|  \|  \|  \|  \|  \|  \|  \|  \|  \|  \|  \|  \| \| Family care \|  \|  \|  \|  \|  \|  \|  \|  \|  \|  \|  \|  \| \| Farming \|  \|  \|  \|  \|  \|  \|  \|  \|  \|  \|  \|  \| \| Agricultural labour for wages \|  \|  \|  \|  \|  \|  \|  \|  \|  \|  \|  \|  \| \| Non-Agricultural labour for wages /Employed work \|  \|  \|  \|  \|  \|  \|  \|  \|  \|  \|  \|  \| \| Self-employed work \|  \|  \|  \|  \|  \|  \|  \|  \|  \|  \|  \|  \| \| Watching TV/reading/ Mobile \|  \|  \|  \|  \|  \|  \|  \|  \|  \|  \|  \|  \| \| Social activities \|  \|  \|  \|  \|  \|  \|  \|  \|  \|  \|  \|  \| \| Shopping \|  \|  \|  \|  \|  \|  \|  \|  \|  \|  \|  \|  \| \| Travelling \|  \|  \|  \|  \|  \|  \|  \|  \|  \|  \|  \|  \| \| Exercising \|  \|  \|  \|  \|  \|  \|  \|  \|  \|  \|  \|  \| \| School/ College \|  \|  \|  \|  \|  \|  \|  \|  \|  \|  \|  \|  \| \| Religious activities \|  \|  \|  \|  \|  \|  \|  \|  \|  \|  \|  \|  \| \| Other \|  \|  \|  \|  \|  \|  \|  \|  \|  \|  \|  \|  \| \| \| --- \| --- \| --- \| --- \| --- \| --- \| --- \| --- \| --- \| --- \| --- \| --- \| --- \| --- \| --- \| --- \| --- \| --- \| --- \| --- \| --- \| --- \| --- \| --- \| --- \| --- \| --- \| --- \| --- \| --- \| --- \| --- \| --- \| --- \| --- \| --- \| --- \| --- \| --- \| --- \| --- \| --- \| --- \| --- \| --- \| --- \| --- \| --- \| --- \| --- \| --- \| --- \| --- \| --- \| --- \| --- \| --- \| --- \| --- \| --- \| --- \| --- \| --- \| --- \| --- \| --- \| --- \| --- \| --- \| --- \| --- \| --- \| --- \| --- \| --- \| --- \| --- \| --- \| --- \| --- \| --- \| --- \| --- \| --- \| --- \| --- \| --- \| --- \| --- \| --- \| --- \| --- \| --- \| --- \| --- \| --- \| --- \| --- \| --- \| --- \| --- \| --- \| --- \| --- \| --- \| --- \| --- \| --- \| --- \| --- \| --- \| --- \| --- \| --- \| --- \| --- \| --- \| --- \| --- \| --- \| --- \| --- \| --- \| --- \| --- \| --- \| --- \| --- \| --- \| --- \| --- \| --- \| --- \| --- \| --- \| --- \| --- \| --- \| --- \| --- \| --- \| --- \| --- \| --- \| --- \| --- \| --- \| --- \| --- \| --- \| --- \| --- \| --- \| --- \| --- \| --- \| --- \| --- \| --- \| --- \| --- \| --- \| --- \| --- \| --- \| --- \| --- \| --- \| --- \| --- \| --- \| --- \| --- \| --- \| --- \| --- \| --- \| --- \| --- \| --- \| --- \| --- \| --- \| --- \| --- \| --- \| --- \| --- \| --- \| --- \| --- \| --- \| --- \| --- \| --- \| --- \| --- \| --- \| --- \| --- \| --- \| --- \| --- \| --- \| --- \| --- \| --- \| --- \| --- \| --- \| --- \| --- \| --- \| --- \| --- \| --- \| --- \| --- \| --- \| --- \| --- \| --- \| --- \| --- \| --- \| --- \| --- \| --- \| --- \| --- \| --- \| --- \| --- \| --- \| --- \| --- \| --- \| --- \| --- \| --- \| --- \| --- \| --- \| --- \| --- \| --- \| --- \| --- \| --- \| --- \| --- \| --- \| --- \| --- \| --- \| --- \| --- \| --- \| --- \| --- \| --- \| --- \| --- \| --- \| --- \| --- \| --- \| --- \| --- \| --- \| --- \| --- \| --- \| --- \| --- \| --- \| --- \| --- \| --- \| --- \| --- \| --- \| --- \| --- \| --- \| --- \| --- \|  \| \| **PART D: TIME USE** \| \| \| \| \| \| \| \| \| \| \| \| \| \| --- \| --- \| --- \| --- \| --- \| --- \| --- \| --- \| --- \| --- \| --- \| --- \| --- \| \| Record a log of the activities for the individual in the last complete 24 hours (from yesterday morning 4 AM to today morning 4 AM). The time intervals are marked in 30-minute intervals and one activity can be marked for each time period. \| \| \| \| \| \| \| \| \| \| \| \| \| \| VARIABLE NAME \| 10:00 to 10:30 PM_Part_D_timeuse \| 10:30 to 11:00 PM_Part_D_timeuse \| 11:00 to 11:30 PM_Part_D_timeuse \| 11:30 to 12:00 PM_Part_D_timeuse \| 12:00 to 12:30 AM_Part_D_timeuse \| 12:30 to 1:00 AM_Part_D_timeuse \| 1:00 to 1:30 AM_Part_D_timeuse \| 1:30 to 2:00 AM_Part_D_timeuse \| 2:00 to 2:30 AM_Part_D_timeuse \| 2:30 to 3:00 AM_Part_D_timeuse \| 3:00 to 3:30 AM_Part_D_timeuse \| 3:30 to 4:00 AM_Part_D_timeuse \| \| Time \| 10:00 to 10:30 PM \| 10:30 to 11:00 PM \| 11:00 to 11:30 PM \| 11:30 to 12:00 PM \| 12:00 to 12:30 AM \| 12:30 to 1:00 AM \| 1:00 to 1:30 AM \| 1:30 to 2:00 AM \| 2:00 to 2:30 AM \| 2:30 to 3:00 AM \| 3:00 to 3:30 AM \| 3:30 to 4:00 AM \| \| Resting \|  \|  \|  \|  \|  \|  \|  \|  \|  \|  \|  \|  \| \| Eating \|  \|  \|  \|  \|  \|  \|  \|  \|  \|  \|  \|  \| \| Personal care \|  \|  \|  \|  \|  \|  \|  \|  \|  \|  \|  \|  \| \| 1. Cooking \|  \|  \|  \|  \|  \|  \|  \|  \|  \|  \|  \|  \| \| 2. Domestic work \|  \|  \|  \|  \|  \|  \|  \|  \|  \|  \|  \|  \| \| 3. Family care \|  \|  \|  \|  \|  \|  \|  \|  \|  \|  \|  \|  \| \| 4. Farming \|  \|  \|  \|  \|  \|  \|  \|  \|  \|  \|  \|  \| \| Agricultural labour for wages \|  \|  \|  \|  \|  \|  \|  \|  \|  \|  \|  \|  \| \| Non-Agricultural labour for wages /Employed work \|  \|  \|  \|  \|  \|  \|  \|  \|  \|  \|  \|  \| \| Self-employed work \|  \|  \|  \|  \|  \|  \|  \|  \|  \|  \|  \|  \| \| Watching TV/reading/ Mobile \|  \|  \|  \|  \|  \|  \|  \|  \|  \|  \|  \|  \| \| Social activities \|  \|  \|  \|  \|  \|  \|  \|  \|  \|  \|  \|  \| \| Shopping \|  \|  \|  \|  \|  \|  \|  \|  \|  \|  \|  \|  \| \| Travelling \|  \|  \|  \|  \|  \|  \|  \|  \|  \|  \|  \|  \| \| Exercising \|  \|  \|  \|  \|  \|  \|  \|  \|  \|  \|  \|  \| \| School/ College \|  \|  \|  \|  \|  \|  \|  \|  \|  \|  \|  \|  \| \| Religious activities \|  \|  \|  \|  \|  \|  \|  \|  \|  \|  \|  \|  \| \| Other \|  \|  \|  \|  \|  \|  \|  \|  \|  \|  \|  \|  \| \| \| --- \| --- \| --- \| --- \| --- \| --- \| --- \| --- \| --- \| --- \| --- \| --- \| --- \| --- \| --- \| --- \| --- \| --- \| --- \| --- \| --- \| --- \| --- \| --- \| --- \| --- \| --- \| --- \| --- \| --- \| --- \| --- \| --- \| --- \| --- \| --- \| --- \| --- \| --- \| --- \| --- \| --- \| --- \| --- \| --- \| --- \| --- \| --- \| --- \| --- \| --- \| --- \| --- \| --- \| --- \| --- \| --- \| --- \| --- \| --- \| --- \| --- \| --- \| --- \| --- \| --- \| --- \| --- \| --- \| --- \| --- \| --- \| --- \| --- \| --- \| --- \| --- \| --- \| --- \| --- \| --- \| --- \| --- \| --- \| --- \| --- \| --- \| --- \| --- \| --- \| --- \| --- \| --- \| --- \| --- \| --- \| --- \| --- \| --- \| --- \| --- \| --- \| --- \| --- \| --- \| --- \| --- \| --- \| --- \| --- \| --- \| --- \| --- \| --- \| --- \| --- \| --- \| --- \| --- \| --- \| --- \| --- \| --- \| --- \| --- \| --- \| --- \| --- \| --- \| --- \| --- \| --- \| --- \| --- \| --- \| --- \| --- \| --- \| --- \| --- \| --- \| --- \| --- \| --- \| --- \| --- \| --- \| --- \| --- \| --- \| --- \| --- \| --- \| --- \| --- \| --- \| --- \| --- \| --- \| --- \| --- \| --- \| --- \| --- \| --- \| --- \| --- \| --- \| --- \| --- \| --- \| --- \| --- \| --- \| --- \| --- \| --- \| --- \| --- \| --- \| --- \| --- \| --- \| --- \| --- \| --- \| --- \| --- \| --- \| --- \| --- \| --- \| --- \| --- \| --- \| --- \| --- \| --- \| --- \| --- \| --- \| --- \| --- \| --- \| --- \| --- \| --- \| --- \| --- \| --- \| --- \| --- \| --- \| --- \| --- \| --- \| --- \| --- \| --- \| --- \| --- \| --- \| --- \| --- \| --- \| --- \| --- \| --- \| --- \| --- \| --- \| --- \| --- \| --- \| --- \| --- \| --- \| --- \| --- \| --- \| --- \| --- \| --- \| --- \| --- \| --- \| --- \| --- \| --- \| --- \| --- \| --- \| --- \| --- \| --- \| --- \| --- \| --- \| --- \| --- \| --- \| --- \| --- \| --- \| --- \| --- \| --- \| --- \| --- \| --- \| --- \| --- \| --- \| --- \| --- \| --- \| --- \| --- \| --- \| --- \| --- \| --- \| --- \| --- \| --- \| --- \| --- \| | | | | |
| **NO.** | **VARIABLE NAME** | **QUESTION** | **OPTIONS** | **RESPONSE** |
| 1. | [QD1] | In the last 24 hours did you work (at home or outside of the home) more than usual, about the same as usual, or less than usual? | More than usual = 1  About the same as usual = 2  Less than usual = 3 |  |
| **DIET QUALITY QUESTIONNAIRE** | | | | |
|  | Read: Now I’d like to ask you some yes-or-no questions about foods and drinks that you consumed yesterday during the day or night, whether you had it at home or somewhere else.  First, I would like you to think about yesterday, from the time you woke up through the night. Think to yourself about the first thing you ate or drank after you woke up in the morning ... Think about where you were when you had any food or drink in the middle of the day ... Think about where you were when you had any evening meal ... and any food or drink you may have had in the evening or late-night... and any other snacks or drinks you may have had between meals throughout the day or night.  I am interested in whether you had the food items I will mention even if they were combined with other foods. Please listen to the list of foods and drinks, and if you ate or drank ANY ONE OF THEM, say yes. | | | |
| Yesterday, did you eat any of the following foods: | | | | |
| 1. | [QD1_1] | Rice, idli, dosa, poha, naan, kulcha, paratha, or upma? | Yes = 1  No = 2 |  |
| 2.1 | [QD2_1] | Chapati, roti, dalia, or roasted maize? | Yes = 1  No = 2 |  |
| 2.2 | [QD2_2] | Pearl millet or finger millet? | Yes = 1  No = 2 |  |
| 3. | [QD3] | Potato, sweet potato, turnip, arum root, tapioca, or raw banana? | Yes = 1  No = 2 |  |
| 4. | [QD4] | Daal, sambar, chickpeas, kidney beans, soya, or khichdi? | Yes = 1  No = 2 |  |
| Yesterday, did you eat any of the following vegetables: | | | | |
| 5. | [QD5] | Carrots, or pumpkin that is orange inside? | Yes = 1  No = 2 |  |
| 6.1 | [QD6_1] | Mustard leaves, spinach, radish leaves, cassava leaves, taro leaves, drumstick leaves, amaranth leaves, or wild greens/other greens? | Yes = 1  No = 2 |  |
| 7.1 | [QD7_1] | Tomatoes, eggplant, okra/lady finger, French beans, cauliflower, cabbage, or beetroot? | Yes = 1  No = 2 |  |
| 7.2 | [QD7_2] | Bitter gourd, bottle gourd, pointed gourd, ivy gourd, apple gourd, ridged gourd, or snake gourd? | Yes = 1  No = 2 |  |
| 7.3 | [QD7_3] | Cucumber, radish, capsicum, German turnip, or drumstick? | Yes = 1  No = 2 |  |
| Yesterday, did you eat any of the following fruits: | | | | |
| 8. | [QD8] | Papaya, mango, orange musk melon, or apricots? | Yes = 1  No = 2 |  |
| 9. | [QD9] | Orange, tangerine, or grapefruit? | Yes = 1  No = 2 |  |
| 10.1 | [QD10_1] | Ripe banana, apple, pear, watermelon, guava, custard apple, pomegranate, or pineapple? | Yes = 1  No = 2 |  |
| 10.2 | [QD10_2] | Grapes, kiwi, peaches, jackfruit, chickoo, jamun, palmyra palm fruit, or other wild fruits? | Yes = 1  No = 2 |  |
| Yesterday, did you eat any of the following sweets: | | | | |
| 11. | [QD11] | Cakes, cream biscuits, biscuits, suji halwa / kesari bath, jalebi, or ladoo? | Yes = 1  No = 2 |  |
| 12. | [QD12] | Other mithai, rice pudding, kulfi, ice cream, milkshake, toffees, or chocolates? | Yes = 1  No = 2 |  |
| Yesterday, did you eat any of the following foods of animal origin: | | | | |
| 13. | [QD13] | Eggs? | Yes = 1  No = 2 |  |
| 14. | [QD14] | Paneer or cheese? | Yes = 1  No = 2 |  |
| 15. | [QD15] | Curd, lassi, buttermilk, or raita? | Yes = 1  No = 2 |  |
| 16. | [QD16] | Are you vegetarian? | Yes = 1[skip to Q 22]  No = 2 |  |
| 17. | [QD17] | Sausages or salami? | Yes = 1  No = 2 |  |
| 18. | [QD18] | Mutton, lamb, or liver? | Yes = 1  No = 2 |  |
| 19. | [QD19] | Pork or wild meat? | Yes = 1  No = 2 |  |
| 20. | [QD20] | Chicken, duck, or turkey? | Yes = 1  No = 2 |  |
| 21. | [QD21] | Fish, prawn, crab, or seafood? | Yes = 1  No = 2 |  |
| Yesterday, did you eat any of the following other foods: | | | | |
| 22. | [QD22] | Peanuts, cashews, almonds, pistachios, walnuts, pumpkin seeds, or sunflower seeds? | Yes = 1  No = 2 |  |
| 23. | [QD23] | Potato chips, namkeen or mixture? | Yes = 1  No = 2 |  |
| 24. | [QD24 | Instant noodles such as Maggi noodles or Wai Wai? | Yes = 1  No = 2 |  |
| 25. | [QD25] | Samosa, pakora, puri, vada, mathri, kachori, murukku, or bonda? | Yes = 1  No = 2 |  |
| Yesterday, did you have any of the following beverages: | | | | |
| 26. | [QD26] | Milk, flavoured milk, chai with milk, or coffee with milk? | Yes = 1  No = 2 |  |
| 27. | [QD27] | Tea with sugar, coffee with sugar, milk with sugar, flavoured milk, Bournevita, Horlicks, or Boost? | Yes = 1  No = 2 |  |
| 28. | [QD28] | Fruit juice, packet juice such as Rasna or Frooti, sugarcane juice, or nannari sarbath? | Yes = 1  No = 2 |  |
| 29. | [QD29] | Soft drinks such as Sprite, Pepsi, Mirinda, or energy drinks? | Yes = 1  No = 2 |  |
| Yesterday, did you get food from any place like….. | | | | |
| 30. | [QD30] | McDonald's, KFC, Pizza Hut, Domino's, Burger King, or other places that serve pizza or burgers? | Yes = 1  No = 2 |  |
| **DECISION MAKING** | | | | |
| **PART A: Role in Household Decision-Making Around Production and Income Generation** | | | | |
| **NO.** | **VARIABLE NAME** | **QUESTION** | **OPTIONS** | **RESPONSE** |
| Activity A. Food Crop Farming (Food Crop are crops grown ONLY for home consumption) | | | | |
| 1. | [PART_A_Q1] | Did you yourself participate in food crop farming in the past 12 months? | Yes = 1  No = 2 [skip to Activity B] |  |
| 2. | [A_PART_A_Q2_1  To  A_PART_A_Q2_5] | When decisions are made regarding food crop farming, who is it that normally makes the decision?  *Hint: Check all that apply.* | Self = 1 [skip to Activity B if self only]  Spouse = 2  Other Household Member = 3  Other Non-Household Member = 4  Not Applicable = 5 [skip to Activity B] |  |
| 3. | [PART_A_Q3] | How much input did you have in making decisions about food crop farming? | No input or input in few decisions = 1  Input into some decisions = 2  Input into all or most decisions = 3  Not Applicable = 4 [skip to Activity B] |  |
| Activity B. Cash Crop Farming (Cash Crop are crops grown for sale in the market) | | | | |
| 4. | [PART_A_Q4] | Did you yourself participate in cash crop farming in the past 12 months? | Yes = 1  No = 2 [skip to Activity C] |  |
| 5. | [A_PART_A_Q5_1  To  A_PART_A_Q5_5] | When decisions are made regarding cash crop farming, who is it that normally makes the decision?  *Hint: Check all that apply.* | Self =1 [skip to Q7 if self only]  Spouse = 2  Other Household Member = 3  Other Non-Household Member = 4  Not Applicable = 5 [skip to Activity C] |  |
| 6. | [PART_A_Q6] | How much input did you have in making decisions about cash crop farming? | No input or input in few decisions = 1  Input into some decisions = 2  Input into all or most decisions = 3  Not Applicable = 4 [skip to Activity C] |  |
| 7. | [PART_A_Q7] | How much input did you have in decisions on the use of income generated from cash crop farming? | No input or input in few decisions = 1  Input into some decisions = 2  Input into all or most decisions = 3  Not Applicable = 4 |  |
| Activity C. Livestock Rearing | | | | |
| 8. | [PART_A_Q8] | Did you yourself participate in livestock rearing in the past 12 months? | Yes = 1  No = 2 [skip to Activity D] |  |
| 9. | [A_PART_A_Q9_1  To  A_PART_A_Q9_5] | When decisions are made regarding livestock rearing, who is it that normally makes the decision?  *Hint: Check all that apply.* | Self =1 [skip to Q11 if self only]  Spouse = 2  Other Household Member = 3  Other Non-Household Member = 4  Not Applicable = 5 [skip to Activity D] |  |
| 10. | [PART_A_Q10] | How much input did you have in making decisions about livestock rearing? | No input or input in few decisions = 1  Input into some decisions = 2  Input into all or most decisions = 3  Not Applicable = 4 [skip to Activity D] |  |
| 11. | [PART_A_Q11] | How much input did you have in decisions on the use of income generated from livestock raising? | No input or input in few decisions = 1  Input into some decisions = 2  Input into all or most decisions = 3  Not Applicable = 4 |  |
| Activity D. Non-Farm Economic Activities (e.g., a small non-agricultural business) | | | | |
| 12. | [PART_A_Q12] | Did you yourself participate in non-farm economic activities in the past 12 months? | Yes = 1  No = 2 [skip to Activity E] |  |
| 13. | [A_PART_A_Q13_1  To  A_PART_A_Q13_5] | When decisions are made regarding non-farm economic activities, who is it that normally makes the decision?  *Hint: Check all that apply.* | Self =1 [skip to Q15 if self only]  Spouse = 2  Other Household Member = 3  Other Non-Household Member = 4  Not Applicable = 5 [skip to Activity E] |  |
| 14. | [PART_A_Q14] | How much input did you have in making decisions about non-farm economic activities? | No input or input in few decisions = 1  Input into some decisions = 2  Input into all or most decisions = 3  Not Applicable = 4 [skip to Activity E] |  |
| 15. | [PART_A_Q15] | How much input did you have in decisions on the use of income generated from non-farm economic activities? | No input or input in few decisions = 1  Input into some decisions = 2  Input into all or most decisions = 3  Not Applicable = 4 |  |
| Activity E. Wage and Salary Employment (e.g., agricultural labour) | | | | |
| 16. | [PART_A_Q16] | Did you yourself participate in waged or salaried labour in the past 12 months? | Yes = 1  No = 2 [skip to Activity F] |  |
| 17. | [A_PART_A_Q17_1  To  A_PART_A_Q17_5] | When decisions are made regarding waged or salaried labour, who is it that normally makes the decision?  *Hint: Check all that apply.* | Self =1 [skip to Q19 if self only]  Spouse = 2  Other Household Member = 3  Other Non-Household Member = 4  Not Applicable = 5 [skip to Activity F] |  |
| 18. | [PART_A_Q18] | How much input did you have in making decisions about waged or salaried labour? | No input or input in few decisions = 1  Input into some decisions = 2  Input into all or most decisions = 3  Not Applicable = 4 [skip to Activity F] |  |
| 19. | [PART_A_Q19] | How much input did you have in decisions on the use of income generated from waged or salaried labour? | No input or input in few decisions = 1  Input into some decisions = 2  Input into all or most decisions = 3  Not Applicable = 4 |  |
| Activity F. Major Household Expenditures (e.g., health care, education, appliances) | | | | |
| 20. | [A_PART_A_Q20_1  To  A_PART_A_Q20_5] | When decisions are made regarding major household expenditures, who is it that normally makes the decision?  *Hint: Check all that apply.* | Self =1 [skip to Activity G if self only]  Spouse = 2  Other Household Member = 3  Other Non-Household Member = 4  Not Applicable = 5 [skip to Activity G] |  |
| 21. | [A_PART_A_Q21] | How much input did you have in making decisions about major household expenditures? | No input or input in few decisions = 1  Input into some decisions = 2  Input into all or most decisions = 3  Not Applicable = 4 |  |
| Activity G. Minor Household Expenditure (e.g., daily needs such as food and household items like soap) | | | | |
| 22. | [A_PART_A_Q22_1 To A_PART_A_Q22_5] | When decisions are made regarding minor household expenditures, who is it that normally makes the decision?  *Hint: Check all that apply.* | Self =1 [skip to Part B if self only]  Spouse = 2  Other Household Member = 3  Other Non-Household Member = 4  Not Applicable = 5 [skip to Part B] |  |
| 23. | [PART_A_Q23] | How much input did you have in making decisions about minor household expenditures? | No input or input in few decisions = 1  Input into some decisions = 2  Input into all or most decisions = 3  Not Applicable = 4 [skip to Part B] |  |
| **Part B: Access to Productive Capital** | | | | |
| **NO.** | **VARIABLE NAME** | **QUESTION** | **OPTIONS** | **RESPONSE** |
| 1. | [Part_B_Q1] | Does anyone in your household currently have any agricultural land? | Yes, I own it solely = 1  Yes, I own it jointly = 2  Yes, I own some solely, some jointly = 3  Yes, someone else in the household owns it = 4  No, household does not own it = 5 [skip to Q4]  I do not know = 6 [skip to Q4] |  |
| 1a. | [T_Part_B_Q1A_1 To  T_Part_B_Q1A_4] | How much agricultural land does your household own? | XX acres  XX cents  XX bigha  XX biswa |  |
| 2. | [Part_B_Q2] | How much say do you have in renting, mortgage or sale of land? | No say = 1  Some = 2  Major = 3  Final/Main decision-maker = 4  Not Applicable = 5 |  |
| 3. | [Part_B_Q3] | In the past one year has it ever happened that earnings from land assets have been used without your consent? | Yes = 1  No = 2 |  |
| 4. | [Part_B_Q4] | Does anyone in your household currently have any large livestock (e.g., cows or buffalo)? | Yes, I own it solely = 1  Yes, I own it jointly = 2  Yes, I own some solely, some jointly = 3  Yes, someone else in the household owns it = 4  No, household does not own it = 5 [skip to Q6] |  |
| 5. | [A_Part_B_Q5_1  To  A_Part_B_Q5_4] | Which large livestock? | Cows = 1  Buffaloes = 2  Bulls = 3  Other = 4 |  |
| 6. | [Part_B_Q6] | Does anyone in your household currently have any small livestock or poultry (e.g., chickens or goats)? | Yes, I own it solely = 1  Yes, I own it jointly = 2  Yes, I own some solely, some jointly = 3  Yes, someone else in the household owns it = 4  No, household does not own it = 5 [skip to Q8] |  |
| 7. | [A_Part_B_Q7_1  To  A_Part_B_Q7_5] | Which small livestock? | Chickens = 1  Goats = 2  Sheep = 3  Pigs = 4  Other = 5 |  |
| 8. | [Part_B_Q8] | Does anyone in your household currently have any Non-mechanised Farm Equipment (e.g., hoe, animal-drawn cart, backpack sprayer)? | Yes, I own it solely = 1  Yes, I own it jointly = 2  Yes, I own some solely, some jointly = 3  Yes, someone else in the household owns it = 4  No, household does not own it = 5 |  |
| 9. | [Part_B_Q9] | Does anyone in your household currently have any Mechanised Farm Equipment (e.g., tractor/mini truck, tiller, thresher, water pump)? | Yes, I own it solely = 1  Yes, I own it jointly = 2  Yes, I own some solely, some jointly = 3  Yes, someone else in the household owns it = 4  No, household does not own it = 5 |  |
| 10. | [Part_B_Q10] | Does anyone in your household currently have any Non-farm Business Equipment (e.g., sewing machine, rice or daal mill, auto-rikshaw)? | Yes, I own it solely = 1  Yes, I own it jointly = 2  Yes, I own some solely, some jointly = 3  Yes, someone else in the household owns it = 4  No, household does not own it = 5 |  |
| 11. | [Part_B_Q11] | Does anyone in your household currently have a house or any other structure? | Yes, I own it solely = 1  Yes, I own it jointly = 2  Yes, I own some solely, some jointly = 3  Yes, someone else in the household owns it = 4  No, household does not own it = 5  I do not know = 6 |  |
| 12. | [Part_B_Q12] | Does anyone in your household currently have any Large Consumer Durables (e.g., TV, air cooler, refrigerator, washing machine, computer/laptop, mattress)? | Yes, I own it solely = 1  Yes, I own it jointly = 2  Yes, I own some solely, some jointly = 3  Yes, someone else in the household owns it = 4  No, household does not own it = 5 |  |
| 13. | [Part_B_Q13] | Does anyone in your household currently have any Small Consumer Durables (e.g., cooking utensils)? | Yes, I own it solely = 1  Yes, I own it jointly = 2  Yes, I own some solely, some jointly = 3  Yes, someone else in the household owns it = 4  No, household does not own it = 5 |  |
| 14. | [Part_B_Q14] | Does anyone in your household currently have a cell phone? | Yes, I own it solely = 1  Yes, I own it jointly = 2  Yes, I own some solely, some jointly = 3  Yes, someone else in the household owns it = 4  No, household does not own it = 5 |  |
| 15. | [Part_B_Q15] | Does anyone in your household currently have any Other Land Not Used for Agricultural Purposes? | Yes, I own it solely = 1  Yes, I own it jointly = 2  Yes, I own some solely, some jointly = 3  Yes, someone else in the household owns it = 4  No, household does not own it = 5  I do not know = 6 |  |
| 16. | [Part_B_Q16] | Does anyone in your household currently have any Means of Transport (e.g., bicycle, motorcycle/scooter, car/jeep)? | Yes, I own it solely = 1  Yes, I own it jointly = 2  Yes, I own some solely, some jointly = 3  Yes, someone else in the household owns it = 4  No, household does not own it = 5 |  |
| **Part C: Access to Credit** | | | | |
| **NO.** | **VARIABLE NAME** | **QUESTION** | **OPTIONS** | **RESPONSE** |
| Lending Source A. Formal Lender (Bank / Financial Institution) | | | | |
| 1. | [Part_C_Q1] | Would you or anyone in your household be able to take a loan or borrow cash/in-kind from a formal lender if you wanted to? | Yes = 1  No = 2 [Skip to lending source B]  Maybe = 3  I do not know = 4 [skip to lending source B] |  |
| 2. | [Part_C_Q2] | Has anyone in your household taken any loans or borrowed cash/in-kind from a formal lender in the past 12 months? | Yes = 1  No = 2 [Skip to lending source B]  Don’t know = 3 |  |
| 3. | [A_Part_C_Q3_1  To  A_Part_C_Q3_5] | Who made the decision to borrow from a formal lender most of the time?  *Hint: Check all that apply.* | Self =1  Spouse = 2  Other Household Member = 3  Other Non-Household Member = 4  Not Applicable = 5 |  |
| 4. | [A_Part_C_Q4_1  To  A_Part_C_Q4_5] | Who makes the decision about what to do with the money / item borrowed from a formal lender most of the time?  *Hint: Check all that apply.* | Self =1  Spouse = 2  Other Household Member = 3  Other Non-Household Member = 4  Not Applicable = 5 |  |
| Lending Source B. Informal Lender (Local Money Lender / Input Shop / Machine Rental Provider) | | | | |
| 5. | [Part_C_Q5] | Would you or anyone in your household be able to take a loan or borrow cash/in-kind from an informal lender if you wanted to? | Yes = 1  No = 2 [Skip to lending source C]  Maybe = 3  I do not know = 4 [skip to lending source C] |  |
| 6. | [Part_C_Q6] | Has anyone in your household taken any loans or borrowed cash/in-kind from an informal lender in the past 12 months? | Yes = 1  No = 2 [Skip to lending source C]  Don’t know = 3 |  |
| 7. | [A_Part_C_Q7_1  To  A_Part_C_Q7_5] | Who made the decision to borrow from an informal lender most of the time?  *Hint: Check all that apply.* | Self =1  Spouse = 2  Other Household Member = 3  Other Non-Household Member = 4  Not Applicable = 5 |  |
| 8. | [A_Part_C_Q8_1  To  A_Part_C_Q8_5] | Who makes the decision about what to do with the money / item borrowed from an informal lender most of the time?  *Hint: Check all that apply.* | Self =1  Spouse = 2  Other Household Member = 3  Other Non-Household Member = 4  Not Applicable = 5 |  |
| Lending Source C. Friends or Relatives | | | | |
| 9. | [Part_C_Q9] | Would you or anyone in your household be able to take a loan or borrow cash/in-kind from friends or relatives if you wanted to? | Yes = 1  No = 2 [Skip to lending source D]  Maybe = 3  I do not know = 4 [skip to lending source D] |  |
| 10. | [Part_C_Q10] | Has anyone in your household taken any loans or borrowed cash/in-kind from friends or relatives in the past 12 months? | Yes = 1  No = 2 [Skip to lending source D]  Don’t know = 3 |  |
| 11. | [A_Part_C_Q11_1  To  A_Part_C_Q11_5] | Who made the decision to borrow from friends or relatives most of the time?  *Hint: Check all that apply.* | Self =1  Spouse = 2  Other Household Member = 3  Other Non-Household Member = 4  Not Applicable = 5 |  |
| 12. | [A_Part_C_Q12_1  To  A_Part_C_Q12_5] | Who makes the decision about what to do with the money / item borrowed from friends or relatives most of the time?  *Hint: Check all that apply.* | Self =1  Spouse = 2  Other Household Member = 3  Other Non-Household Member = 4  Not Applicable = 5 |  |
|  | Lending Source D. Group-Based Micro-finance or lending including SHGs | | | |
| 13. | [Part_C_Q13] | Would you or anyone in your household be able to take a loan or borrow cash/in-kind from a group based micro- finance or lending source if you wanted to? | Yes = 1  No = 2 [skip to Part D]  Maybe = 3  I do not know = 4 [skip to part D] |  |
| 14. | [Part_C_Q14] | Has anyone in your household taken any loans or borrowed cash/in-kind from a group based micro- finance or lending source in the past 12 months? | Yes = 1  No = 2 [skip to Part D]  Don’t know = 3 |  |
| 15. | [A_Part_C_Q15_1  To  A_Part_C_Q15_5] | Who made the decision to borrow from a group based micro- finance or lending source most of the time?  *Hint: Check all that apply.* | Self =1  Spouse = 2  Other Household Member = 3  Other Non-Household Member = 4  Not Applicable = 5 |  |
| 16. | [A_Part_C_Q16_1  To  A_Part_C_Q16_5] | Who makes the decision about what to do with the money / item borrowed from a group based micro- finance or lending source most of the time?  *Hint: Check all that apply.* | Self =1  Spouse = 2  Other Household Member = 3  Other Non-Household Member = 4  Not Applicable = 5 |  |
|  | **Part D: Group Membership** | | | |
| **NO.** | **VARIABLE NAME** | **QUESTION** | **OPTIONS** | **RESPONSE** |
| 1. | [Part_D_Q1] | Is there a FPO in your community? | Yes = 1  No = 2 [skip to Q3]  Don’t know = 3 [skip to Q3] |  |
| 2. | [Part_D_Q2] | Are you an active member of the FPO? | Yes = 1  No = 2 |  |
|  | Asked to Women Only | | | |
| 3. | [Part_D_Q3] | Is there a SHG in your community? | Yes = 1  No = 2 [skip to PART E]  Don’t know = 3 [skip to PART E] |  |
| 4. | [Part_D_Q4] | Are you an active member of the SHG? | Yes = 1  No = 2 [skip to PART E] |  |
| 5. | [Part_D_Q5] | What is your role in the SHG? | SHG President = 1  SHG bookkeeper = 2  SHG Vice President = 3  SHG Secretary = 4  Other = 5 |  |
| 6. | [Part_D_Q6] | How comfortable do you feel sharing your opinion or asking questions in the SHG? | Very uncomfortable = 1  Uncomfortable = 2  Neither comfortable nor uncomfortable = 3  Comfortable = 4  Very comfortable = 5 |  |
|  | **PART E: Physical Mobility** | | | |
| **NO.** | **VARIABLE NAME** | **QUESTION** | **OPTIONS** | **RESPONSE** |
| 1. | [Part_E_Q1] | How often do you visit an urban center? | Daily = 1  Weekly = 2  Every 2 weeks = 3  Monthly = 4  Every couple months = 5  Less than every 2 months = 6  Never = 7 |  |
| 2. | [Part_E_Q2] | How often do you go to the market? | Daily = 1  Weekly = 2  Every 2 weeks = 3  Monthly = 4  Every couple months = 5  Less than every 2 months = 6  Never = 7 |  |
| 3. | [Part_E_Q3] | How often have you visited a bank or post office alone in order to conduct any business? | Never = 1  A few times = 2  Many times = 3 |  |
| 4. | [Part_E_Q4] | How often do you go to visit family or relatives? | Daily = 1  Weekly = 2  Every 2 weeks = 3  Monthly = 4  Every couple months = 5  Less than every 2 months = 6  Never = 7 |  |
| 5. | [Part_E_Q5] | How often do you go to visit a friend / neighbor’s house? | Daily = 1  Weekly = 2  Every 2 weeks = 3  Monthly = 4  Every couple months = 5  Less than every 2 months = 6  Never = 7 |  |
| 6. | [Part_E_Q6] | How often do you go to the hospital / clinic / doctor to seek health services for your own healthcare? | Daily = 1  Weekly = 2  Every 2 weeks = 3  Monthly = 4  Every couple months = 5  Less than every 2 months = 6  Never = 7 |  |
| 7. | [Part_E_Q7] | How often do you go to a public gathering (e.g.: community meeting / training for NGO / SHG meeting or programs)? | Daily = 1  Weekly = 2  Every 2 weeks = 3  Monthly = 4  Every couple months = 5  Less than every 2 months = 6  Never = 7 |  |
| 8. | [Part_E_Q8] | In the last 12 months, how many times have you been away from home for one or more nights (in other words, sleeping somewhere else for the night)? | Daily = 1  Weekly = 2  Every 2 weeks = 3  Monthly = 4  Every couple months = 5  Less than every 2 months = 6  Never = 7 |  |
| 9. | [Part_E_Q9] | In the last 12 months, have you been away from home for more than one month at a time? | Yes = 1  No = 2 |  |
| **SECTIONS BELOW TO BE ASKED ONLY TO MALE PARTICIPANTS IN THE SURVEY** | | | | |
| **HOUSEHOLD ECONOMICS** | | | | |
| **The following questions on income refer to total income at the household level** | | | | |
| **NO.** | **VARIABLE NAME** | **QUESTION** | **OPTIONS** | **RESPONSE** |
| 1. | [HE_Q1] | Total number of members living in the household including respondent.  *Hint: “residing in this household” means a group of people who eat from the same kitchen.* | XX number | [min 1, max 20] |
| 2. | [HE_Q2] | Did you have income from **cultivation** in the past 12 months? | Yes = 1  No = 2 [skip to Q4] |  |
| 3. | [HE_Q3] | What was the total earned from cultivation in the past 12 months? | XX Rupees (₹) | [min 1] |
| 4. | [HE_Q4] | Did you have income from **livestock** in the past 12 months? | Yes = 1  No = 2 [skip to Q6] |  |
| 5. | [HE_Q5] | What was the total earned from livestock in the past 12 months? | XX Rupees (₹) | [min 1] |
| 6. | [HE_Q6] | Did you have income from **other agricultural activity** in the past 12 months? For example, selling fodder, fisheries, bee keeping, silk worms. | Yes = 1  No = 2 [skip to Q8] |  |
| 7. | [HE_Q7] | What was the total earned from other agricultural activity in the past 12 months? | XX Rupees (₹) | [min 1] |
| 8. | [HE_Q8] | Did you have income from **non-agricultural enterprises** in the past 12 months? | Yes = 1  No = 2 [skip to Q10] |  |
| 9. | [HE_Q9] | If yes, what was the total earned from this source in the last 12 months? | XX Rupees (₹) | [min 1] |
| 10. | [HE_Q10] | Did you have income from **wages** in the past 12 months? | Yes = 1  No = 2 [skip to Q12] |  |
| 11. | [HE_Q11] | If yes, what was the total earned from this source in the last 12 months? | XX Rupees (₹) | [min 1] |
| 12. | [HE_Q12] | Did you have income from **salaried employment** in the past 12 months? | Yes = 1  No = 2 [skip to Q14] |  |
| 13. | [HE_Q13] | If yes, what was the total earned from this source in the last 12 months? | XX Rupees (₹) | [min 1] |
| Expenditure | | | | |
| 14. | [HE_Q14] | During the last week (7 days), how much money did you spend on food for your household, including food eaten at home and food eaten away from home? | XX Rupees (₹) | [min 1] |
| **AGRICULTURAL PRACTICES** | | | | |
| **Part A: For Market Sales and home consumption** | | | | |
| **NO.** | **VARIABLE NAME** | **QUESTION** | **OPTIONS** | **RESPONSE** |
| 1. | [T_AP_Q1_1 To T_AP_Q1_4] | How much land did you cultivate in Kharif (monsoon) 2022? | XX acres (1)  XX cents (2)  XX bigha (3)  XX biswa (4) |  |
| 2. | [AP_Q2] | How many crops did you grow in Kharif (monsoon) 2022?    *Consider all vegetables grown on one plot to be one crop.* | Number |  |
| 3. | [AP_Q3] | Did you cultivate the same amount of land in Rabi (winter) 2022? | Yes = 1 [skip to Q5]  No = 2 |  |
| 4. | [T_AP_Q4_1  To  T_AP_Q4_4] | How much land did you cultivate in Rabi (winter) 2022? | XX acres (1)  XX cents (2)  XX bigha (3)  XX biswa (4)  [skip to Q6 if 0] |  |
| 5. | [AP_Q5] | How many crops did you cultivate in Rabi (winter) 2022? | Number |  |
| Begin loop for each crop up to 5 crops. Please indicate the major crops grown by the farmer in terms of area cultivated in the past year (include both Kharif and Rabi crops). | | | | |
| 6. | [A_AP_Q6_1  To  A_AP_Q6_28] | Crop type | Maize = 1  Paddy = 2  Wheat = 3  Jowar = 4  Foxtail millet = 5  Finger millet = 6  Little millet = 7  Kodo millet = 8  Proso millet = 9  Barnyard millet = 10  Pearl millet = 11  Redgram = 12  Blackgram = 13  Green Gram = 14  Chickpea = 15  Cowpea = 16  Groundnut = 17  Sunflower = 18  Castor = 19  Sesame =20  Fruit (specify) = 21 [Add Q10 only if fruits opted]  Vegetable (specify) = 22 [Add Q8 only if fruits opted]               Flower crop = 23  Cotton = 24  Sugarcane =25  Coriander = 26  Mushroom = 27  Mixed Vegetables = 29  Other (specify) = 28 [Add Q7 if other opted] |  |
| 7. | [S_AP_Q6_28] | Specify Other Crops | Text |  |
| 8. | [A_AP_Q8_1  To  A_AP_Q8_18] | Specify which vegetables    *Check all that apply.* | Red chili = 1  Onion = 2  Tomato = 3  Lemon = 4  Cluster bean = 5  Ladyfinger = 6  Drumstick = 7  Eggplant = 8  Bitter gourd = 9  Bottle gourd = 10  English cucumber = 11  Cucumber = 12  Pumpkin = 13  Ash gourd = 14  Leafy vegetables = 15  Green Peas = 16  Field beans = 17  Other (specify) = 18 [Add Q9 if other opted] |  |
| 9. | [S_AP_Q8_18] | Specify other vegetables | Text |  |
| 10. | [AP_Q10_O1  To  AP_Q10_O12] | Specify which fruits    *Check all that apply.* | Banana = 1  Mango = 2  Guava = 3  Sapota = 4  Sweet lime = 5  Pomegranate = 6  Papaya = 7  Neredu (Jamun) = 8  Custard apple = 9  Watermelon = 10  Strawberry = 11  Other (specify) = 12 [Add Q11 if other opted] |  |
| 11. | [S_AP_Q10_12] | Specify other fruits | Text |  |
| 12. |  | What is the area under this crop? | XXX acres  XXX cents  XXX bigha  XX biswa |  |
|  |  | Select 5 crops for loop formation.  Hint: Please select top 5 (maximum) crops based on the land area cultivated (for market sales and home consumption). | The crops previously selected would appear here. |  |
| 13. | [Maize_AP_Q13] | Did you finish harvesting this crop? | Yes, fully harvested = 1  Yes, at least one full harvest = 2  No = 3 [skip to Part B] |  |
| 14. | [Maize_A_AP_Q14_1  To  Maize_A_AP_Q14_12] | In what month did you harvest this crop? | January = 1  February = 2  March = 3  April = 4  May = 5  June = 6  July = 7  August = 8  September = 9  October = 10  November = 11  December = 12 |  |
| 15. | [Maize_AP_Q15] | What was the total yield for this crop? | XX |  |
| 16. | [Maize_AP_Q16] | Unit? | Per acre = 1 [answer Q17]  per week = 2 [answer Q17]  total = 3 [answer Q17]  bags = 4 [answer Q18] |  |
| 17. | [Maize_AP_Q17] | kg or quintal? | kg = 1 [answer Q20]  quintal = 2 [answer Q20] |  |
| 18. | [Maize_AP_Q18] | How many kg per bag? | 50 kg = 1  75 kg = 2  80 kg = 3  Other (specify) = 4 [answer Q19] |  |
| 19. | [Maize_Q_204_S] | Specify other quantity per bag | XX kg |  |
| 20. | [Maize_AP_Q20] | Was the product sold? | Sold Fully = 1 [Skip to Q22]  Sold Partially = 2  Not sold at all = 3 [answer Q21 and skip to Part B] |  |
| 21. | [Maize_A_AP_Q21_1  To  Maize_A_AP_Q21_8] | Reason not sold.  (Regarding the produce that was not sold partially or fully)  *Check all that apply.* | Home consumption = 1  Farm gate losses = 2  Losses during transport = 3  Trader did not accept all produce = 4  Turned into value-added product = 5  Stored for later sale = 6  Stored for seeds = 7  Other = 8 |  |
| 22. | [Maize_AP_Q22] | Quantity of product sold  *Hint: This includes sales to all buyers in whatever form.* | XX |  |
| 23. | [Maize_AP_Q23] | Unit of product sold? | kg= 1 [answer Q27]  quintal = 2[answer Q27]  bags = 3 [answer Q25]  other unit (specify) = 4 [answer Q24] |  |
| 24. | [Maize_Q_208_S] | Other unit of product sold | Text [answer Q27] |  |
| 25. | [Maize_AP_Q25] | How many kg per bag? | 50 kg = 1  75 kg = 2  80 kg = 3  Other (specify) = 4[answer Q26] |  |
| 26. | [Maize_Q_209_S] | Specify other quantity per bag | XX kg |  |
| 27.  27. | [Maize_AP_Q27] | Total amount received for the product sold  *Hint: This is the total amount across all transactions.* | XX Rupees (₹) |  |
| 28. | [Maize_A_AP_Q28_1  To  Maize_A_AP_Q28_7] | Where was the product sold?  *Check all that apply.* | Village Market = 1  At government market/mandi = 2  To trader = 3  FPO = 4 [Skip Part B if FPO not selected]  Krishi Melas = 5  Directly to consumer = 6  Other = 7 |  |
| 29. | [Maize_AP_Q29] | What quantity of the product was sold at FPO? | XX |  |
| 30. | [Maize_AP_Q30] | Unit of the quantity of product sold at FPO | kg= 1 [answer Q34]  quintal = 2 [answer Q34]  bags = 3 [answer Q32]  other unit (specify) = 4[answer Q31] |  |
| 31. | [Maize_Q_213_S] | Other units of product sold at FPO | Text [answer 34] |  |
| 32. | [Maize_AP_Q32] | How many kg per bag? | 50 kg = 1  75 kg = 2  80 kg = 3  Other (specify) = 4[answer Q33 if other selected] |  |
| 33. | [Maize_Q_214_S] | Specify other quantity per bag | XX kg [answer Q34] |  |
| 34. | [Maize_AP_Q34] | Total amount received for the product sold at FPO. | XX Rupees (₹) |  |
| **Part B: FPO and access to inputs and technical knowledge** | | | | |
| Ask to both FPO and control villages | | | | |
| 35. | [AP_Q35] | Did you regularly receive any farm related advisories in the past year (12 months)? | Yes = 1  No = 2 [skip to Q38] |  |
| 36. | [A_AP_Q36_1  To  A_AP_Q36_1] | What are the types of advisories received?  *Check all that apply* | Weather = 1  Pest incidence and management = 2  Market prices = 3  Other = 4 |  |
| 37. | [A_AP_Q37_1  To  A_AP_Q37_4] | Who provided these advisories?  *Check all that apply* | FPO = 1  Government or KVK (Krishi Vigyan Kendra) = 2  FPO in collaboration with government = 3  Other organization = 4 |  |
| 38. | [AP_Q38] | Did you receive any training on crop production in the past year(12 months)? | Yes = 1  No = 2 [Skip to Q40] |  |
| 39. | [AP_Q39_O1  To  AP_Q39_O4] | Who provided this training?  *Check all that apply* | FPO = 1  Government/KVK = 2  FPO in collaboration with government = 3  Other organization = 4 |  |
| Ask to FPO members only [make conditional on “FPO HOUSEHOLD” to Part A Q5] | | | | |
| 40. | [AP_Q40] | Did you purchase any inputs from the FPO in the past year (12 months)? | Yes = 1  No = 2 [Skip to Q42] |  |
| 41. | [A_AP_Q41_1 To  A_AP_Q41_4] | What input did you purchase?  *Check all that apply* | Fertilizer = 1  Pesticide = 2  Seeds = 3  Other = 4 |  |
| 42. | [AP_Q42] | Did you use any of the processing facilities of the FPO in the past year (12 months) on a rental basis? | Yes = 1  No = 2 [End Survey] |  |
| 43. | [AP_Q43_O1  To  AP_Q43_O4] | What processing facilities of the FPO did you use in the past year (12 months) on a rental basis?  *Check all that apply* | Daal mill = 1  Jaggerry making unit = 2  Biomass-based cold storage/Ripening chamber = 3  storage facility = 4  [End Survey] |  |
