# Supplementary material for: Association of membership in a farmer producer organization with crop diversity, household income, diet diversity, and women’s empowerment in Uttar Pradesh, India
Source: PLoS One. 2025 Mar 26;20(3):e0319704. doi: 10.1371/journal.pone.0319704 (PMC11940710; doi:10.1371/journal.pone.0319704)
Supplement: Attachment — Submitted filename: Response to Reviewers.docx [file pone.0319704.s006.docx]

*We thank the reviewers for their time and careful consideration of our manuscript. We have incorporated their suggestions, which we believe have improved the manuscript. We provide point-by-point responses below highlighting changes made in the text. Line numbers below refer to the clean version of the manuscript (without tracked changes).*

**Reviewer #1:**

Overall comments: This well-designed mixed-methods study provides valuable insights into FPO impacts on agricultural households in Uttar Pradesh. The research demonstrates robust methodology and statistical analysis, though causal relationships cannot be established. Findings have important implications for agricultural and nutrition policy.

*Response: Thank you for taking the time to review our manuscript and for recognizing the importance of this study. We appreciate your feedback, to which we have responded inline below.*

1. The study reports that "FPO households had slightly greater crop production diversity than non-FPO households (mean of 4 crops versus 3.5 crops, respectively)." How meaningful is this small difference in practical terms for agricultural sustainability and household nutrition?

*Response: We agree with the reviewer that this difference, while statistically significant (p=0.005), is not likely to be nutritionally significant. We have prefaced all statements regarding this difference with “slightly” and have expanded our discussion on the implications of a difference of this magnitude in the revised manuscript. (p. 21, lines 377-383)*

*“This effect size is comparable to, for example, a recent program implemented by Bangladesh’s Ministry of Agriculture which had the explicit aim of increasing production diversity (effect size of 0.3-0.4 crops) [39], but evidence from previous studies suggests that much larger increases in on-farm crop diversity are needed to impact nutritional outcomes such as diet diversity [40]. However, smaller increases in on-farm crop diversity—for example, comparing one crop (monocropping) to more than one crop—may be associated with enhanced biodiversity [41].”*

2. The observed pattern of "lower sale prices for FPO households selling to traders" versus "higher prices for non-FPO households selling to village markets" appears counterintuitive given that FPOs are meant to improve market access. This warrants deeper investigation into market dynamics.

*Response: We agree with the reviewer that this warrants deeper investigation into market dynamics in future studies and have added this point to the revised discussion. However, we caution against over-interpretation or generalization of these findings from our analysis given that less than half of households in our sample sold any of their produce and a very small percentage (<4%) had fully sold their produce (Table 2). The sale price is therefore only available for a small percentage of the sample (17% of FPO and non-FPO households cultivating wheat, 9% of FPO households cultivating paddy, and 7% of non-FPO households cultivating paddy). For this reason, we did not conduct any formal statistical testing of differences between FPO and non-FPO households for this exploratory analysis. The mean differences are small (~163 INR/quintal for paddy and ~119 INR/quintal for wheat compared to SDs of 449-525 INR/quintal). We therefore now preface all statements regarding this difference with “slightly”. (pp. 23-24, lines 415-431)*

*“We found that most households—both FPO and non-FPO—in this sample did not sell their paddy or wheat. It is possible that some farmers who were planning to sell their produce had not yet sold it, as data were collected between 28 April and 30 May. Among those who did sell their produce, which was less than half of the sample and so should be cautiously interpreted, FPO households sold slightly greater quantities, but received slightly lower prices than non-FPO households—possibly because they sold to traders more frequently than non-FPO households who were more likely to sell to village markets. However, the differences were small (~163 INR/quintal for paddy and ~119 INR/quintal for wheat) and could reflect reporting errors rather than true differences in sale price. The proportion of both FPO and non-FPO households selling at government markets was relatively low, especially for paddy (only 3.4% of FPO households and 6.5% of non-FPO households sold paddy to government markets), and on average, households received a price less than or only slightly more than the minimum support price (MSP) for paddy and wheat [43]: MSP 2,183 for common paddy in 2023/24 versus mean sale price of 1,491 for FPO households and 1,654 for non-FPO households and MSP 2,125 for wheat in 2023/24 versus mean sale price of 2,022 for FPO households and 2,142 for non-FPO households. Future research should focus on investigating these market dynamics for FPO versus non-FPO households.”*

3. "While we adjusted for educational attainment, caste, and farm size, FPO and non-FPO households may have differed with regards to other characteristics not captured by the survey" - Could you identify specific unmeasured variables that might confound the relationships observed?

*Response: Thank you for this suggestion. We have listed examples of other characteristics not captured by the survey that could be considered in future studies to the revised discussion. (pp. 25-26, lines 473-477)*

*“For example, previous studies have found that access to Internet, primary occupation in farming, years of farming experience, contact with agricultural extension workers or receipt of information from government agricultural agencies, and farmers’ intentions (e.g., to expand operations) may influence FPO membership [17–19,21].”*

4. The study indicates mixed effects on diet quality, with increased consumption of both healthy and unhealthy foods in FPO households. What specific nutrition education interventions could be integrated into FPO programs to promote healthier dietary choices?

*Response: Thank you for suggesting this. We have expanded our discussion of evidence-based policy options to promote healthier dietary choices in the revised manuscript. (pp. 20-21 lines 358-362 & p. 24 lines 444-452)*

*“Such policies should consider integrating FPO support measures such as postharvest technologies to introduce affordable nutrient-rich foods for year-round sale in rural markets, as well as implementing a national tax on high fat, sugar, and salt products given evidence that health taxes represent an effective win-win for public health and public finances [34–36].”*

*“Previous studies suggest that nutrition behavior change communication (BCC) to groups such as self-help groups in India has a limited effect on nutritional status, particularly when it is an under-resourced add-on intervention and does not have a sustained high intensity of implementation [46–50]. Moreover, nutrition-sensitive agriculture programs with a BCC component tend to focus on undernutrition and promoting healthy foods, rather than discouraging unhealthy foods (often, consumption of unhealthy foods is not even assessed) [46,48–50]. Thus, policies should instead consider changes to the food environment such as the introduction of affordable nutrient-rich foods for year-round sale in rural markets and a national tax on high fat, sugar, and salt products [34–36].”*

5. "Only 10% of FPO households purchased any inputs from the FPO in the past year" suggests very low utilization of FPO services. What are the barriers preventing members from taking advantage of these resources?

*Response: As this study was an impact evaluation, unfortunately, we did not collect process evaluation information such as specific barriers faced by individual FPO members. We have highlighted this as a key gap to be addressed by future research in the revised discussion. (p. 21, lines 362-373)*

*“Two important gaps for further research include (1) a deeper understanding of the challenges faced by FPOs, including market dynamics as well as barriers faced by the farmers in availing of FPO services, and (2) testing which specific services delivered to FPO members are most effective for improving outcomes such as income, nutrition, empowerment, and sustainability. FPOs can provide a number of services, including facilitating access to credit, providing storage and processing facilities, reducing the cost of inputs (through economies of scale), increasing bargaining power, opening doors to new markets, and/or delivering training relating to agricultural practices, new technologies, value-add activities, business, management, etc. [37,38]. Evidence suggests it may be better to focus on providing a few services well [38], which would require further research to understand which services would be most effective for which outcomes across different contexts.”*

6. The statement that "28% of FPO households reported receiving farm-related advisories in the past year" seems quite low given that extension services are a key benefit of FPO membership. How could advisory outreach be improved?

*Response: As this study was an impact evaluation, unfortunately, we did not collect process evaluation information such as specific improvements that could be made to increase access and utilization of FPO services. We have highlighted this as a key gap to be addressed by future research in the revised discussion (please see response to comment #5 for new text added to the revised manuscript).*

7. The research notes income discrepancies: "The latest Situation Assessment Survey...found average monthly income was 8,061 INR... more than twice the income reported by households in our sample." Does this suggest potential methodological issues in income measurement?

*Response: The survey instrument used to assess household income in our study was adapted from the survey instrument used in the latest Situation Assessment Survey of Agricultural Households. Nonetheless, as the reviewer points out, it is possible that there was misreporting, particularly under-reporting of income in our study. However, it is more likely that the income discrepancy stems from the fact that our sample population is poorer than the state average. We have clarified this, citing data from the latest round of the National Family Health Survey (2019-2021), in the revised discussion. (pp. 22-23, lines 400-407)*

*“The context of our study, Fatehpur district, also has a lower economic status than the state of Uttar Pradesh, on average. For example, according to the latest National Family Health Survey (2019-2021), only 77% of the population in Fatehpur is living in households with electricity, 63% use an improved sanitation facility, and 39% use clean fuel for cooking compared to 91%, 69%, and 50% of households, respectively, at the state level [23]. Female literacy rates in the district are also lower than the state average: 62% versus 66%, respectively [23]. These factors could also partially explain the lower income observed in our sample as compared to the state average.”*

8. While the study employed mixed methods, the qualitative component involving only three stakeholder interviews seems limited. Could expanding qualitative research provide deeper insights into the challenges and opportunities?

*Response: Thank you for this suggestion. The “mixed methods” aspect of this study included the household survey, the market basket survey (direct observation of all open and operating vendors in 13 villages) as well as the semi-structured qualitative interviews with key stakeholders (the CEO of the FPO,* *a representative of the state agriculture department, and a district-level government administrative official). While we agree with the reviewer that further qualitative interviews may provide additional insights into the challenges and opportunities for FPOs, this was outside the scope of the current study—these interviews were conducted in order to provide further context for interpreting the results of the household surveys.* *As one of the first studies in India to empirically evaluate the impact of FPOs, we feel this project will open doors for further qualitative research as a next step, and we highlight this point in the revised discussion. (p. 21, lines 362-373)*

*“Two important gaps for further research include (1) a deeper understanding of the challenges faced by FPOs, including market dynamics as well as barriers faced by the farmers in availing of FPO services, and (2) testing which specific services delivered to FPO members are most effective for improving outcomes such as income, nutrition, empowerment, and sustainability.”*

9. Given the finding that "access to and decisions about agricultural credit and self-help group membership were particularly low among both FPO and non-FPO women," what specific policy interventions could improve women's access to financial resources?

*Response: We have added further detail to the revised discussion regarding what specific interventions are available to improve women’s access to credit in the Indian context. (p. 25, lines 460-467)*

*“Several reasons have been proposed for low credit access among women in rural India including inactive accounts [51] and low engagement (financial institutions often give credit based on an applicant’s transaction history); lack of collateral due to limited access to assets and property; distance to financial institutions; and lack of women in frontline roles in the financial sector [52,53]. Financial and digital literacy programs for women, enabling the use of mobile technology to access accounts and credit, gender-sensitive innovations in credit scoring for women farmers [54], and increasing access to collateral-free loans could help address these barriers.”*

10. The study could be improved by integrating insights from recent research in related fields: <https://doi.org/10.3390/ma15207098>; <https://doi.org/10.3390/ma15186222>.

*Response: We think there may have been an error in the DOIs provided as these refer to studies on materials science (“Fly Ash-Based Geopolymer Composites: A Review of the Compressive Strength and Microstructure Analysis” and “Concrete Containing Waste Glass as an Environmentally Friendly Aggregate: A Review on Fresh and Mechanical Characteristics”), which are not relevant to our study on farmer producer organizations in India.*

**Reviewer #3:**

Overall comments: Manuscript PONE-D-24-46385 presents famer producer organizations (FPOs), a kind of member farmer cooperatives’ socio-economic impacts on income, crop diversity, and household livelihoods of the local farmers of a state of India. Research on membership of FPOs and their socio-economic impacts on the farmers in developing countries substantially reported in the literature and the manuscript does not sufficiently differentiate novel research aspects from existing literature for a clear articulation of the current findings’ contribution towards significantly advancement of the existing knowledge. The two outcomes of the membership of FPOs viz., women’s empowerment and dietary diversity adds bit of novelty, however; there is lack of in-depth exploration of the mechanisms through which FPO membership influences dietary patterns and gender dynamics of the women diversity in FPO, which put a limitation to the innovative contribution of the given study to nutrition-sensitive agriculture. Limited critical analyses of results on women’s empowerment which show no significant differences between FPO and non-FPO households. The study is based on limited sample size i.e., only a single FPO which put a limitation to generate broadly applicable insights and uncover unique patterns related to comparative analyses across different FPOs, regions, or organizational structures. The causal inferences related to FPO’s emerging benefits and impacts over time are extremely limited as the given study is cross-sectional in nature. The distinct theoretical and methodological framework lacking e.g., combining of the standard tools like the FAO minimum diet diversity score and A-WEAI index for generating a deeper unexpected insight of the FPO’s dynamics. The policy recommendation implications are general and are like the recommendations given in multiple studies. The detailed comments are as under:

*Response: Thank you for taking the time to review our manuscript. We have responded to each of the above-mentioned comments and detailed comments point-by-point below. With regards to the novelty of the study, our literature review highlighted that most research on farmer cooperatives has been conducted in high-income countries, and nearly no studies have evaluated key farmer-centric outcomes—beyond yield and income—in a developing country context such as dietary diversity (as an indicator for nutrition) or women’s empowerment. We have expanded our discussion of underlying mechanisms for these outcomes in the revised manuscript. While we agree with and note in our manuscript the limitations the reviewer points out, namely the cross-sectional nature of the study and relatively small sample size (though our sample size was larger than a couple previous studies on FPOs in India, e.g., our sample was n=819 households compared to n=560 households in a Sikkim-based FPO study* [*https://doi.org/10.1108/IJSE-04-2022-0216*](https://doi.org/10.1108/IJSE-04-2022-0216)*, n=300 households in a Gujarat-based study* [*http://dx.doi.org/10.5958/0974-0279.2019.00023.5*](http://dx.doi.org/10.5958/0974-0279.2019.00023.5)*, and n=550 households in a Bihar-based study* [*http://dx.doi.org/10.5958/0974-0279.2019.00022.3*](http://dx.doi.org/10.5958/0974-0279.2019.00022.3)*), this is the first impact evaluation of these outcomes in the Indian context, where FPOs are being promoted at scale across the country. Our hope is that findings from this novel pilot study will encourage concurrent impact and process evaluations in future programs and schemes as well as randomized controlled trials to improve causal estimation. Thank you again for your kind support of this work.*

Title:

1. The title is too long, jargon, complex, and lack with impact orientation with imbalanced emphasis on the novel aspects of the study. The tile requires significant improvement. An alternative form could be “Membership of farmer producer organizations in Uttar Pradesh (India): Effects on Agriculture, Diet, and Women’s Empowerment.”

*Response: Thank you for this suggestion. We have shortened the title and removed jargon such as “mixed methods”. However, we would prefer to retain “association of” rather than “effects on” because we feel “effects on” implies causality and we do not want to over-state our findings. The PLOS One guidelines (*[*https://journals.plos.org/plosone/s/submission-guidelines*](https://journals.plos.org/plosone/s/submission-guidelines)*) require the title to be specific and under 250 characters, and the revised title is 162 characters (with spaces), so well within the author guidelines.*

Abstract:

2. The contextual background about specific outcomes of farmer cooperative in the Indian and global context is crucial, which may clearly be described in the abstract.

*Response: We have been asked by the Editor to adhere to all PLOS One guidelines (*[*https://journals.plos.org/plosone/s/submission-guidelines*](https://journals.plos.org/plosone/s/submission-guidelines)*) including for the abstract to be under 300 words. We have therefore had to cut 56 words from our original abstract and were not able to add these further details. Nonetheless, we agree these are important details to include in the manuscript and have included them in the revised manuscript’s introduction.*

3. The integration of qualitative components viz., dietary diversity and women empowerment in the mixed methods require imbalance in terms of establishing causal relationships between farmer cooperatives and the selected qualitative variables.

*Response: We have been asked by the Editor to adhere to all PLOS One guidelines (*[*https://journals.plos.org/plosone/s/submission-guidelines*](https://journals.plos.org/plosone/s/submission-guidelines)*) including for the abstract to be under 300 words. We have therefore had to cut 56 words from our original abstract and were not able to add these further details. We felt it was most important to include a sentence on results for each of the four primary outcomes (crop diversity, household income, diet diversity, and women’s empowerment) to align with our stated objectives. The mixed methods (e.g., market basket survey and qualitative interviews) were conducted as secondary objectives, to add context to facilitate interpretation of the findings for the four primary outcomes.*

4. Discerning which findings are most significant in the given results may be revisited.

*Response: We have included a sentence on results for each of the four primary outcomes (crop diversity, household income, diet diversity, and women’s empowerment) to align with our stated objectives.*

5. There is lack of nuanced discussion on limitations of the study and conclusion are highly generic.

*Response: We have been asked by the Editor to adhere to all PLOS One guidelines (*[*https://journals.plos.org/plosone/s/submission-guidelines*](https://journals.plos.org/plosone/s/submission-guidelines)*) including for the abstract to be under 300 words. We have therefore had to cut 56 words from our original abstract and were not able to add these further details. Nonetheless, we agree these are important details to include and have included them in the revised manuscript’s discussion.*

6. Inclusion of GA [graphical abstract] is suggested.

*Response: We have been asked by the Editor to adhere to all PLOS One guidelines (*[*https://journals.plos.org/plosone/s/submission-guidelines*](https://journals.plos.org/plosone/s/submission-guidelines)*) including for the abstract and so could not include a graphical abstract.*

Keywords:

7. For enhanced discoverability of the draft (in case it gets published), include keywords that are and specifically relevant to the study rather than being highly general.

*Response: We have carefully reviewed our keywords relative to other studies published in PLOS One and other studies published on this topic to ensure they are specific enough to facilitate identification, for example, we have used “nutrition-sensitive agriculture” instead of just “agriculture”.*

Highlights:

8. Inclusion of highlights is suggested.

*Response: We have been asked by the Editor to adhere to all PLOS One guidelines (*[*https://journals.plos.org/plosone/s/submission-guidelines*](https://journals.plos.org/plosone/s/submission-guidelines)*) and so could not include highlights.*

Introduction:

9. There is lack of in-depth summarizing of the existing research related to the specific variables being studied in the given manuscript. As the study is a kind of case study of a state of India, mentioning studies of other countries like Uganda, Ethiopia, etc. doesn’t related to the specific sate context of Uttar Perdesh. Include a critical discussion of India’s FPO-related policies, such as the government’s 2020 initiative to form 10,000 FPOs, and explore gaps in their implementation. Contextualize local FPO-related information with the famer cooperative effectiveness in improving agricultural and socio-economic outcomes of the developed countries. A clear dentification and description of the existing gaps in the literature is missing. Background related to the women empowerment in the context of FPOs requires significant improvement. There is quite a missed opportunity to explain methodological rationale and critical analyses of the limitations and challenges related to implementation hurdles and regional disparities.

*Response: We have been asked by the Editor to adhere to all PLOS One guidelines (*[*https://journals.plos.org/plosone/s/submission-guidelines*](https://journals.plos.org/plosone/s/submission-guidelines)*) which state that the introduction should “Include a brief review of the key literature”. While we agree that findings from studies in Uganda, Ethiopia, [Europe], etc. may not be directly generalizable to the Indian context, we felt these studies needed to be briefly mentioned as they are the only impact evaluations of farmer cooperatives to assess women’s empowerment as far as we are aware from our literature review. No such studies exist in India, hence why the study we present in this manuscript is so novel. By leaving these studies out, we would not be accurately reflecting the full body of evidence regarding impacts of farmer cooperatives. To address the reviewer’s comment, we have greatly expanded the discussion of previous literature relating to FPOs in India, in the revised introduction. (p. 5, lines 86-92)*

*“In this context, the impact of FPOs is under-explored in the empirical literature [16–21]. A few studies have found FPO membership to be associated with higher incomes [17,19]. For example, a study comparing 274 FPO households to 276 non-FPO households in Bihar found that, on average, FPO household income was ~Rs.2,200/month more than non-FPO households [17]. Likewise, a study comparing 200 FPO households to 100 non-FPO households in Gujarat found that, on average, FPO household income was ~Rs.778/month more than non-FPO households [19].”*

Methods:

10. The claimed use of mixed-methods approach lacks with sufficient description of integration of qualitative and quantitative data. No description provided about the specific contribution of the qualitative variables (i.e., interviews) and the derived and analysed data. Any coding and/or thematic analyses fo the qualitative interviews not described, which compromise rigor of the used method instrument.

*Response: Thank you for this suggestion. The “mixed methods” aspect of this study included the household survey, the market basket survey (direct observation of all open and operating vendors in 13 villages) as well as the semi-structured qualitative interviews with key stakeholders (the CEO of the FPO,* *a representative of the state agriculture department, and a district-level government administrative official). While we agree with the reviewer that further qualitative interviews may provide additional insights into the challenges and opportunities for FPOs, this was outside the scope of the current study—these interviews were conducted in order to provide further context for interpreting the results of the household surveys.* *As one of the first studies in India to empirically evaluate the impact of FPOs, we feel this project will open doors for further qualitative research as a next step, and we highlight this point in the revised discussion. (p. 21, lines 362-373)*

*“Two important gaps for further research include (1) a deeper understanding of the challenges faced by FPOs, including market dynamics as well as barriers faced by the farmers in availing of FPO services, and (2) testing which specific services delivered to FPO members are most effective for improving outcomes such as income, nutrition, empowerment, and sustainability.”*

11. Sample limitations are unaddressed in the draft e.g., quota sampling may inherently carry biasness since it is non-random and potentially impact on the generalizability of the given data.

*Response: We agree with the reviewer and have added this limitation of the sampling strategy to the revised discussion (p. 26, lines 480-482).*

*“Moreover, we used quota sampling rather than random sampling, which may have introduced sampling bias. Thus, our findings may not be generalizable to all FPOs in Uttar Pradesh or elsewhere in India.”*

12. The survey instruments like A-WEAI and FAO diet diversity score lack with description of specific modifications adopted in the current study.

*Response: Thank you for pointing out this missing detail in our methods. We have added further description of the modifications made to the A-WEAI in the revised methods. We did not modify the FAO diet diversity score. (p. 8, lines 156-162)*

*“The slight modifications to the A-WEAI were made in order to reduce participant burden and specifically included: (1) collecting time use in 30-minute intervals instead of 15-minute intervals and (2) not asking, “To what extent do you feel you can make your own personal decisions regarding [ACTIVITY] if you want(ed) to?” as part of the decision-making module because respondents in pilot testing were unable to distinguish between this question and the question, “How much input did you have in making decisions about [ACTIVITY]?” [30].”*

13. Potential impacts of the extraneous variables like seasonal effects are overlooked. Confounding variables like market access, weather variability, etc. are not accounted for.

*Response: Thank you for pointing out this missing detail in our methods. We purposefully selected control (non-FPO) households to be geographically adjacent to FPO households to control through the sampling design for factors such as weather, pest attacks, etc. We have added this detail to the revised methods. (p. 7, lines 131-134)*

*“Quantitative household-level surveys were conducted across 27 villages in the district: 22 villages where FPO members lived and 5 nearby villages without FPO members (herein “control villages”). Nearby villages were purposefully selected to control for potential confounding factors such as weather.”*

14. Details required about how missing data were handled during regression analyses.

*Response: Thank you for pointing out this missing information in our revised methods. We have added a sentence to explain that all analyses were complete-case analyses and added a table to the supporting information summarizing the % missing each variable. In most cases, there was no missing data. In the case of female’s educational attainment, this was missing for n=19 (2.3%), caste was missing for n=1 (<0.1%), and agricultural land ownership was missing for n=14 (1.7%). (p. 12, lines 251-253)*

*“Complete case analysis was used to handle missing data (<10% data missing for all variables, see Table 1 in S1 Text).”*

15. On what criteria did the authors select villages and vendors, and how findings were integrated with household-level data.

*Response: 13 of the 27 villages where participants lived were surveyed. All 5 control villages were selected. The 8 FPO villages were selected based on distance from the FPO office (within ~10 km radius) and availability of shops. This detail has been added to the revised methods. We did not sample vendors, we did a census of all open and operating vendors in these villages. Market basket survey results were not integrated with household-level data as this was outside the scope of the study and we did not seek ethics approval to collect GPS information for participants’ households. (p. 8, lines 165-168)*

*“A market basket survey was conducted on 4 and 5 April 2023 through direct observation of all open and operating vendors in 13 of the 27 villages where participants lived. All 5 control villages were selected. The 8 FPO villages were selected based on distance from the FPO office (within ~10 km radius) and availability of shops.”*

Results:

16. A general impression is that there is overwhelming volume of data that is densely packed with information making it difficult to discern the most critical findings. Results may be prioritized that are directly relevant to the study’s objectives which shifting less critical data to the appendices/supplementary files. The data carries an inconsistent depth of analyses like market access, selling practices, etc. are underexplored.

*Response: We have carefully reviewed the results to ensure that the depth of analyses and results presented are consistent with the stated primary and secondary objectives. The primary objective of this study was to test whether FPO households have greater crop diversity and higher cultivation income, and whether adults in those households have greater diet diversity and women are more empowered (this is stated in the introduction). These four outcomes are consistent with the outcomes stated in the title of the manuscript, in the abstract, and the sub-headings of the results. As stated in the introduction, the secondary objectives of this study were (1) to assess the market availability of nutrient-dense produce in villages with and without FPO members and (2) to understand existing FPO support services and perceived challenges to founding and operating FPOs in this context. To address the reviewer’s comment, we have moved the results on availability of nutrient dense foods to a new results sub-section, before the sub-section on FPO support and challenges to better match the stated secondary objectives. (p. 19, lines 323-333)*

17. Full regression model outputs, confidence intervals, and effect sizes may be clearly given for the regression models used in the current study.

*Response: All regression model outputs including the effect size, SE, 95% confidence interval, and p-value are provided in the Supporting Information S1 Text.*

18. The dietary diversity results lack depth as they do not delve into the significance of higher unhealthy food consumption among FPO households or its implications for nutrition-sensitive agriculture.

*Response: We did not collect data on the health implications of higher unhealthy food consumption in this study (i.e., diabetes, hypertension, etc.) and so cannot comment on it in the results, which focuses on presenting findings based on our data and does not extrapolate beyond what we observed in our data. We do, however, expand further on the implications in the discussion. (pp. 20-21 lines 358-362 & p. 24 lines 444-452)*

*“Such policies should consider integrating FPO support measures such as postharvest technologies to introduce affordable nutrient-rich foods for year-round sale in rural markets, as well as implementing a national tax on high fat, sugar, and salt products given evidence that health taxes represent an effective win-win for public health and public finances [34–36].”*

*“Previous studies suggest that nutrition behavior change communication (BCC) to groups such as self-help groups in India has a limited effect on nutritional status, particularly when it is an under-resourced add-on intervention and does not have a sustained high intensity of implementation [46–50]. Moreover, nutrition-sensitive agriculture programs with a BCC component tend to focus on undernutrition and promoting healthy foods, rather than discouraging unhealthy foods (often, consumption of unhealthy foods is not even assessed) [46,48–50]. Thus, policies should instead consider changes to the food environment such as the introduction of affordable nutrient-rich foods for year-round sale in rural markets and a national tax on high fat, sugar, and salt products [34–36].”*

19. No subgroup analyses provided, such as variations by caste, gender, landholding size, which however, could have deep impact of FPO membership.

*Response: We appreciate the suggestion. We have added results for individual-level outcomes (diet diversity and women’s empowerment) by caste to the Supporting Information S1 Text. Results by gender are included in Figure 3. However, for household-level outcomes (crop diversity and income), we were not able to conduct subgroup analyses by landholding size given the small number of landless, medium, or large farmers (96% of the sample were small farmers). We have added this as a limitation to the revised discussion. (p. 26, lines 482-487)*

*“Finally, we were not able to conduct subgroup analyses by landholding size given the small number of medium and large farms in our sample (96% of our sample was small farms). Future studies should evaluate whether impacts vary across small, medium, and large farms given evidence from previous studies that farms of all sizes may not benefit equally from membership in cooperatives [2,3].”*

Discussion:

20. The analyses of women’s empowerment are quite superficial as there is lack of significant differences in women’s empowerment between FPO and non-FPO households. Socio-cultural barriers, structural challenges, or the role of FPO governance could be examined to better contextualize this result.

*Response: Thank you for this comment. We have expanded our discussion of the challenges faced by women to better contextualize this result, focusing on the findings relating to access to credit. (p. 25, lines 460-467)*

*“Several reasons have been proposed for low credit access among women in rural India including inactive accounts [51] and low engagement (financial institutions often give credit based on an applicant’s transaction history); lack of collateral due to limited access to assets and property; distance to financial institutions; and lack of women in frontline roles in the financial sector [52,53]. Financial and digital literacy programs for women, enabling the use of mobile technology to access accounts and credit, gender-sensitive innovations in credit scoring for women farmers [54], and increasing access to collateral-free loans could help address these barriers.”*

21. The mechanisms by which FPO membership improves outcomes like crop and diet diversity are not discussion in sufficient detail e.g., the role of improved market access or knowledge sharing is underexplored.

*Response: Thank you for this suggestion. We have added a discussion of the role of market access to the paragraphs unpacking the results for crop and diet diversity (p. 22 lines 385-388 & p. 24 lines 435-438)*

*“Many factors influence farmers’ decisions to grow certain crops, including market access, agroclimatic conditions, farmers’ knowledge and experience, and access to inputs, among other factors [42].”*

*“Previous studies in India have found that improving market access and income diversification are critical pathways for improving diet diversity in agricultural households, and mixed effects for the importance of crop diversification, depending on the context [44,45].”*

22. Explanations may be given relate to challenges faced by FPO members or the specific support services provided by FPOs.

*Response: We have added detail regarding the specific support services provided by FPOs to the revised discussion. (p. 21 lines 366-373 & p. 26 lines 488-497)*

*“FPOs can provide a number of services, including facilitating access to credit, providing storage and processing facilities, reducing the cost of inputs (through economies of scale), increasing bargaining power, opening doors to new markets, and/or delivering training relating to agricultural practices, new technologies, value-add activities, business, management, etc. [37,38]. Evidence suggests it may be better to focus on providing a few services well [38], which would require further research to understand which services would be most effective for which outcomes across different contexts.”*

*“In order to achieve the stated goals and other co-benefits of FPOs, the emphasis should not just be on the quantity of FPOs but also the quality of services provided. Such services could include input purchasing, agricultural machinery leasing, value-addition and processing (product differentiation), marketing, market linkages including for export, more reliable contracts, and finance, among others. However, a single FPO cannot be effective at providing all of these services. As a next step, it will be important to understand what services are most important and have the biggest impact on farmers’ income, diet quality, and women’s empowerment in a given context. Collection of information regarding the services provided by registered FPOs should be considered by the Ministry of Corporate Affairs and other FPO portals as such data are currently lacking and are essential for developing effective policies to improve FPO performance [15].”*

23. The dietary implications of the study are not fully addressed e.g., critical analyses of the mixed dietary findings—greater diversity alongside increased unhealthy food consumption—are not provided. The role of income allocation, market access, or dietary behaviour changes due to FPO membership could be discussed further.

*Response: Thank you for suggesting this. We have expanded our discussion of evidence-based policy options to promote healthier dietary choices in the revised manuscript. (pp. 20-21 lines 358-362 & p. 24 lines 444-452)*

*“Such policies should consider integrating FPO support measures such as postharvest technologies to introduce affordable nutrient-rich foods for year-round sale in rural markets, as well as implementing a national tax on high fat, sugar, and salt products given evidence that health taxes represent an effective win-win for public health and public finances [34–36].”*

*“Previous studies suggest that nutrition behavior change communication (BCC) to groups such as self-help groups in India has a limited effect on nutritional status, particularly when it is an under-resourced add-on intervention and does not have a sustained high intensity of implementation [46–50]. Moreover, nutrition-sensitive agriculture programs with a BCC component tend to focus on undernutrition and promoting healthy foods, rather than discouraging unhealthy foods (often, consumption of unhealthy foods is not even assessed) [46,48–50]. Thus, policies should instead consider changes to the food environment such as the introduction of affordable nutrient-rich foods for year-round sale in rural markets and a national tax on high fat, sugar, and salt products [34–36].”*

24. Policy recommendations lack specific relevance to the barriers to women’s empowerment or promoting healthy dietary behaviours amongst the member farmers. A more explicit connection could be built with the global development goals, such as SDGs, particularly related to poverty, gender equality, and nutrition.

*Response: We have added further detail to the revised discussion regarding what specific interventions are available to improve women’s empowerment (p. 25, lines 460-467, see response to comment #20) and healthy diets (pp. 20-21 lines 358-362 & p. 24 lines 444-452, see response to comment #23). We have also added a remark on the connection to the SDGs. (p. 27, lines 503-506)*

*“Together, these actions may accelerate progress towards achieving several UN Sustainable Development Goals in India, including especially no poverty (#1), zero hunger (#2), good health and well-being (#3), gender equality (#5), and decent work and economic growth (#8).”*

Conclusion:

25. Overall, the given conclusion is broad and does not provide specific, actionable recommendations based on the study’s findings e.g., how FPOs can be better supported or what specific interventions could enhance their impact on women’s empowerment. Suggestion may be given related to addressing the gap about lack of significant impact on women’s empowerment, an already acknowledged component of the study.

*Response: We have been asked by the Editor to adhere to all PLOS One guidelines (*[*https://journals.plos.org/plosone/s/submission-guidelines*](https://journals.plos.org/plosone/s/submission-guidelines)*) and have removed the separate “Conclusions” section accordingly to align with a “mixed Discussion/Conclusions section (commonly labeled “Discussion”)”. As such, specific, actionable recommendations are integrated throughout this “mixed Discussion/Conclusions” section.*

26. The conclusion highlights the improvement in diet diversity but does not address the increased consumption of unhealthy foods among FPO households, which is a critical finding requiring further discussion.

*Response: We have been asked by the Editor to adhere to all PLOS One guidelines (*[*https://journals.plos.org/plosone/s/submission-guidelines*](https://journals.plos.org/plosone/s/submission-guidelines)*) and have removed the separate “Conclusions” section accordingly to align with a “mixed Discussion/Conclusions section (commonly labeled “Discussion”)”. As such, the increased consumption of unhealthy foods among FPO households is discussed in detail in this “mixed Discussion/Conclusions” section (pp. 20-21 lines 358-362 & p. 24 lines 444-452, see response to comment #23).*

27. The limitations of the study may be clearly acknowledged especially cross-sectional design and single FPO focus.

*Response: We have been asked by the Editor to adhere to all PLOS One guidelines (*[*https://journals.plos.org/plosone/s/submission-guidelines*](https://journals.plos.org/plosone/s/submission-guidelines)*) and have removed the separate “Conclusions” section accordingly to align with a “mixed Discussion/Conclusions section (commonly labeled “Discussion”)”. As such, the limitations of cross-sectional design and single FPO focus are discussed in detail in this “mixed Discussion/Conclusions” section (p. 25, lines 468-470).*

References:

28. Obsolete references in the Introduction and Discussion may be replaced with the latest ones.

*Response: We have carefully reviewed all references to ensure we are citing the most relevant and recent articles to support statements made in the introduction and discussion.*

**Journal requirements:**

1. Please ensure that your manuscript meets PLOS ONE's style requirements, including those for file naming.

*Response: We confirm that our manuscript meets PLOS ONE's style requirements, including those for file naming.*

2. Please include a complete copy of PLOS’ questionnaire on inclusivity in global research in your revised manuscript. Our policy for research in this area aims to improve transparency in the reporting of research performed outside of researchers’ own country or community. The policy applies to researchers who have travelled to a different country to conduct research, research with Indigenous populations or their lands, and research on cultural artefacts. The questionnaire can also be requested at the journal’s discretion for any other submissions, even if these conditions are not met. Please find more information on the policy and a link to download a blank copy of the questionnaire here: https://journals.plos.org/plosone/s/best-practices-in-research-reporting. Please upload a completed version of your questionnaire as Supporting Information when you resubmit your manuscript.

*Response: We have included a completed version of PLOS’ questionnaire on inclusivity in global research with our revised manuscript and added the following sentence to the ‘Ethics’ sub-section of the revised methods:*

*“Additional information regarding the ethical, cultural, and scientific considerations specific to inclusivity in global research is included in the Supporting Information (S1 Checklist).”*

3. In the ethics statement in the Methods, you have specified that verbal consent was obtained. Please provide additional details regarding how this consent was documented and witnessed, and state whether this was approved by the IRB.

*Response: Verbal consent was documented in the survey software (question 6, the survey is provided in the Supplemental Material). This approach was approved by the IRB. The IRB-approved consent protocol is pasted below:*

*“Surveys will be conducted only after obtaining informed verbal consent from participants. Participants will be given a hard copy of the Participant Information Sheet. Consent will be discussed and given in the participant's native language (Hindi). Consent will be administered by a trained field staff member who is fluent in this language. We may interview individuals with low literacy including those who cannot read. A trained enumerator will slowly and carefully read the Participant Information Sheet out loud, pausing after each section to allow the participant to ask clarifying questions. We will make sure to explain things in clear and simple language. Participants will be told that at any time they can ask if they do not understand a question, and we will explain it or rephrase it. Participants will also be told that they do not have to answer questions if they do not wish to, and that they can stop participating at any time without consequence.”*

*We have added these further details to the ‘Ethics’ sub-section of the revised methods:*

*“All participants were given a hard copy of a Participant Information Sheet describing, using clear and simple language, what would happen if they took part in the study, procedures relating to confidentiality and anonymity, participants’ rights, benefits and risks, and contact details for further information. A trained enumerator slowly and carefully read the Participant Information Sheet out loud, pausing after each section to allow the participant to ask clarifying questions. Informed oral verbal consent was obtained from all participants before proceeding with the household survey or qualitative interview. Verbal consent was documented directly in the survey software (the survey is provided in the Supplemental Material).”*

4. In the online submission form, you indicated that your data will be submitted to a repository upon acceptance. We strongly recommend all authors deposit their data before acceptance, as the process can be lengthy and hold up publication timelines. Please note that, though access restrictions are acceptable now, your entire minimal dataset will need to be made freely accessible if your manuscript is accepted for publication. This policy applies to all data except where public deposition would breach compliance with the protocol approved by your research ethics board. If you are unable to adhere to our open data policy, please kindly revise your statement to explain your reasoning and we will seek the editor's input on an exemption.

*Response:* *There are no ethical or legal restrictions on sharing a de-identified data set. We have uploaded anonymized datasets to the Harvard Dataverse and included an updated Data Availability section in our cover letter including the DOI.*

*“The anonymized dataset necessary to replicate our study findings is available on the Harvard Dataverse at: https://doi.org/10.7910/DVN/QWQRS7*

*Code files necessary to replicate our study findings are available on GitHub at: https://github.com/snishmeet1699/FPO-Evaluation-India”*

5. We note that you have included the phrase “data not shown” in your manuscript. Unfortunately, this does not meet our data sharing requirements. PLOS does not permit references to inaccessible data. We require that authors provide all relevant data within the paper, Supporting Information files, or in an acceptable, public repository. Please add a citation to support this phrase or upload the data that corresponds with these findings to a stable repository (such as Figshare or Dryad) and provide and URLs, DOIs, or accession numbers that may be used to access these data. Or, if the data are not a core part of the research being presented in your study, we ask that you remove the phrase that refers to these data.

*Response: We have removed this phrase as the data are not a core part of the research being presented in our study. We have also uploaded anonymized datasets to the Harvard Dataverse and included an updated Data Availability section in our cover letter including the DOI. This dataset includes individual-level information on all reported income sources should readers be interested in seeing income data further disaggregated.*

6. Please include captions for your Supporting Information files at the end of your manuscript, and update any in-text citations to match accordingly. Please see our Supporting Information guidelines for more information: <http://journals.plos.org/plosone/s/supporting-information>.

*Response: We have added captions for the Supporting Information files to the end of the manuscript and updated in-text citations accordingly.*
