# Supplementary material for: Association of membership in a farmer producer organization with crop diversity, household income, diet diversity, and women’s empowerment in Uttar Pradesh, India
Source: PLoS One. 2025 Mar 26;20(3):e0319704. doi: 10.1371/journal.pone.0319704 (PMC11940710; doi:10.1371/journal.pone.0319704)
Supplement: Attachment — Submitted filename: Reveiwers comments on PONE-D-24-46385.docx [file pone.0319704.s004.docx]

**Reviewer’s comments on** **PONE-D-24-46385**

**Overall comments:** Manuscript PONE-D-24-46385 presents famer producer organizations (FPOs), a kind of member farmer cooperatives’ socio-economic impacts on income, crop diversity, and household livelihoods of the local farmers of a state of India. Research on membership of FPOs and their socio-economic impacts on the farmers in developing countries substantially reported in the literature and the manuscript does not sufficiently differentiate novel research aspects from existing literature for a clear articulation of the current findings’ contribution towards significantly advancement of the existing knowledge. The two outcomes of the membership of FPOs viz., women’s empowerment and dietary diversity adds bit of novelty, however; there is lack of in-depth exploration of the mechanisms through which FPO membership influences dietary patterns and gender dynamics of the women diversity in FPO, which put a limitation to the innovative contribution of the given study to nutrition-sensitive agriculture. Limited critical analyses of results on women’s empowerment which show no significant differences between FPO and non-FPO households. The study is based on limited sample size i.e., only a single FPO which put a limitation to generate broadly applicable insights and uncover unique patterns related to comparative analyses across different FPOs, regions, or organizational structures. The causal inferences related to FPO’s emerging benefits and impacts over time are extremely limited as the given study is cross-sectional in nature. The distinct theoretical and methodological framework lacking e.g., combining of the standard tools like the FAO minimum diet diversity score and A-WEAI index for generating a deeper unexpected insight of the FPO’s dynamics. The policy recommendation implications are general and are like the recommendations given in multiple studies. The detailed comments are as under:

**Title:** The title is too long, jargon, complex, and lack with impact orientation with imbalanced emphasis on the novel aspects of the study. The tile requires significant improvement. An alternative form could be “Membership of farmer producer organizations in Uttar Pradesh (India): Effects on Agriculture, Diet, and Women’s Empowerment.”

**Abstract:** The contextual background about specific outcomes of farmer cooperative in the Indian and global context is crucial, which may clearly be described in the abstract. The integration of qualitative components viz., dietary diversity and women empowerment in the mixed methods require imbalance in terms of establishing causal relationships between farmer cooperatives and the selected qualitative variables. Discerning which findings are most significant in the given results may be revisited. There is lack of nuanced discussion on limitations of the study and conclusion are highly generic.

**Graphical abstract:** Inclusion of GA is suggested.

**Keywords:** For enhanced discoverability of the draft (in case it gets published), include keywords that are and specifically relevant to the study rather than being highly general.

**Highlights:** Inclusion of highlights is suggested.

**Introduction:** There is lack of in-depth summarizing of the existing research related to the specific variables being studied in the given manuscript. As the study is a kind of case study of a state of India, mentioning studies of other countries like Uganda, Ethiopia, etc. doesn’t related to the specific sate context of Uttar Perdesh. Include a critical discussion of India’s FPO-related policies, such as the government’s 2020 initiative to form 10,000 FPOs, and explore gaps in their implementation. Contextualize local FPO-related information with the famer cooperative effectiveness in improving agricultural and socio-economic outcomes of the developed countries. A clear dentification and description of the existing gaps in the literature is missing. Background related to the women empowerment in the context of FPOs requires significant improvement. There is quite a missed opportunity to explain methodological rationale and critical analyses of the limitations and challenges related to implementation hurdles and regional disparities.

**Methods:** The claimed use of mixed-methods approach lacks with sufficient description of integration of qualitative and quantitative data. No description provided about the specific contribution of the qualitative variables (i.e., interviews) and the derived and analysed data. Any coding and/or thematic analyses fo the qualitative interviews not described, which compromise rigor of the used method instrument. Sample limitations are unaddressed in the draft e.g., quota sampling may inherently carry biasness since it is non-random and potentially impact on the generalizability of the given data. The survey instruments like A-WEAI and FAO diet diversity score lack with description of specific modifications adopted in the current study. Potential impacts of the extraneous variables like seasonal effects are overlooked. Confounding variables like market access, weather variability, etc. are not accounted for. Details required about how missing data were handled during regression analyses. On what criteria did the authors select villages and vendors, and how findings were integrated with household-level data.

**Results:** A general impression is that there is overwhelming volume of data that is densely packed with information making it difficult to discern the most critical findings. Results may be prioritized that are directly relevant to the study’s objectives which shifting less critical data to the appendices/supplementary files. The data carries an inconsistent depth of analyses like market access, selling practices, etc. are underexplored. Full regression model outputs, confidence intervals, and effect sizes may be clearly given for the regression models used in the current study. The dietary diversity results lack depth as they do not delve into the significance of higher unhealthy food consumption among FPO households or its implications for nutrition-sensitive agriculture. No subgroup analyses provided, such as variations by caste, gender, landholding size, which however, could have deep impact of FPO membership.

**Discussion:** The analyses of women’s empowerment are quite superficial as there is lack of significant differences in women’s empowerment between FPO and non-FPO households. Socio-cultural barriers, structural challenges, or the role of FPO governance could be examined to better contextualize this result. The mechanisms by which FPO membership improves outcomes like crop and diet diversity are not discussion in sufficient detail e.g., the role of improved market access or knowledge sharing is underexplored. Explanations may be given relate to challenges faced by FPO members or the specific support services provided by FPOs. The dietary implications of the study are not fully addressed e.g., critical analyses of the mixed dietary findings—greater diversity alongside increased unhealthy food consumption—are not provided. The role of income allocation, market access, or dietary behaviour changes due to FPO membership could be discussed further. Policy recommendations lack specific relevance to the barriers to women’s empowerment or promoting healthy dietary behaviours amongst the member farmers. A more explicit connection could be built with the global development goals, such as SDGs, particularly related to poverty, gender equality, and nutrition.

**Conclusion:** Overall, the given conclusion is broad and does not provide specific, actionable recommendations based on the study’s findings e.g., how FPOs can be better supported or what specific interventions could enhance their impact on women’s empowerment. Suggestion may be given related to addressing the gap about lack of significant impact on women’s empowerment, an already acknowledged component of the study. The conclusion highlights the improvement in diet diversity but does not address the increased consumption of unhealthy foods among FPO households, which is a critical finding requiring further discussion. The limitations of the study may be clearly acknowledged especially cross-sectional design and single FPO focus.

**References:** Obsolete references in the Introduction and Discussion may be replaced with the latest ones.
